# Drawing a high-resolution functional map of adeno-associated virus capsid by massively parallel sequencing

**DOI:** 10.1038/ncomms4075

**Published:** 2014-01-17

**Authors:** Kei Adachi, Tatsuji Enoki, Yasuhiro Kawano, Michael Veraz, Hiroyuki Nakai

**Affiliations:** 1 Department of Molecular and Medical Genetics, Oregon Health and Science University School of Medicine, Portland, Oregon 97239, USA; 2 Takara Bio Inc., Otsu, Shiga 520-2134, Japan; 3 A portion of the study was conducted at University of Pittsburgh School of Medicine, to which K.A., T.E. and H.N. belonged until June 2011

## Abstract

Adeno-associated virus (AAV) capsid engineering is an emerging approach to advance gene therapy. However, a systematic analysis on how each capsid amino acid contributes to multiple functions remains challenging. Here we show proof-of-principle and successful application of a novel approach, termed AAV Barcode-Seq, that allows us to characterize phenotypes of hundreds of different AAV strains in a high-throughput manner and therefore overcomes technical difficulties in the systematic analysis. In this approach, we generate DNA barcode-tagged AAV libraries and determine a spectrum of phenotypes of each AAV strain by Illumina barcode sequencing. By applying this method to AAV capsid mutant libraries tagged with DNA barcodes, we can draw a high-resolution map of AAV capsid amino acids important for the structural integrity and functions including receptor binding, tropism, neutralization and blood clearance. Thus, Barcode-Seq provides a new tool to generate a valuable resource for virus and gene therapy research.

## Abstract (web summary)

Adeno-associated viruses (AAV) are promising gene transfer vectors for human gene therapy. Here, the authors report a high-throughput, sequencing-based method for characterizing amino acid function in AAV capsids and highlight the potential of this method for improving capsid design.

## Graphical abstract

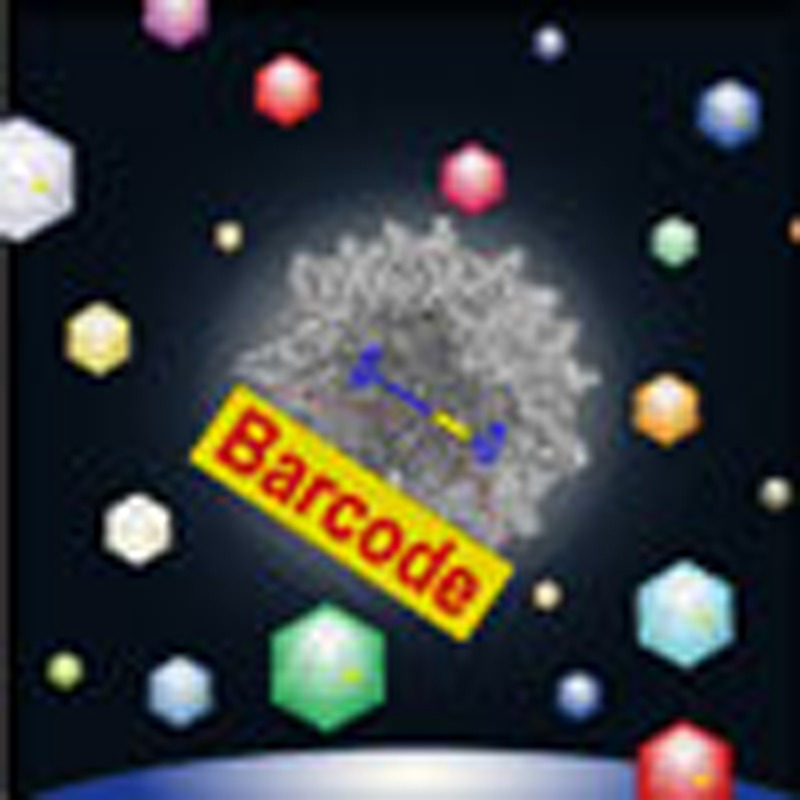

Adeno-associated virus (AAV) is an attractive gene delivery vector for human gene therapy. However, various issues remain to be overcome, including the requirement of high vector dose for clinically beneficial outcomes^1^,^2^, efficacy-limiting host immune responses against viral proteins^1^,^2^, promiscuous viral tropism and the high prevalence of pre-existing anti-AAV neutralizing antibodies in humans^3^,^4^. Despite these issues, enthusiasm and hope for the use of AAV vectors in gene therapy is growing. This is owed in part to the amenability of the capsids to genetic manipulation for the creation of novel targeting vectors and vectors with a stealth phenotype^5^. A series of site-directed mutagenesis studies^6^,^7^,^8^,^9^,^10^,^11^,^12^,^13^,^14^,^15^,^16^,^17^,^18^ and the elucidation of the atomic structures of the prototype AAV serotype 2 (AAV2)^19^ and other serotypes^20^,^21^,^22^,^23^ have provided insights into the structural basis of the AAV capsid functions. Such conventional approaches have helped identify amino-acid residues that play roles in binding to cell surface receptors^7^,^8^,^24^,^25^,^26^ and neutralizing antibodies^27^,^28^. They also assisted in designing more potent AAV capsids by surface-exposed tyrosine residue mutations^29^,^30^,^31^,^32^ and those with selective tropism by re-engineering a cell surface receptor footprint^33^. However, structural knowledge-based prediction of viral capsid functions remains a significant challenge. Directed evolution approaches have recently become increasingly common in the development of novel AAV capsids that target specific cell types with an enhanced efficiency^34^,^35^,^36^,^37^. This approach does not require prior knowledge but relies on the power of iterative positive selection from a pool of diverse mutants. Mutants selected by this method, however, often suffer from a lack of structural and functional interpretation of phenotypes, which limits the use of evolved amino-acid sequence information for the development of new logical approaches.

Here, we develop a novel next-generation sequencing (NGS)-based method, termed AAV Barcode-Seq. This new method utilizes a DNA barcode-tagged mutagenesis approach in conjunction with multiplexed Illumina sequencing. We report proof-of-principle and successful application of this method to comprehensively identify the structural and functional roles of a total of 381 amino acids in the entire carboxy (C)-terminal half of the AAV9 capsid and a total of ~70 amino acids within the largest loop in the AAV1, AAV6, AAV7, AAV8 and AAV9 VP capsid proteins. In addition, we present two successful cases that utilized the new knowledge obtained from AAV Barcode-Seq data to design and create new AAV capsids with directed phenotypes. Barcode-Seq is a new approach that significantly advances virus and gene therapy research.

## Results

### Experimental design

**Figure 1:**
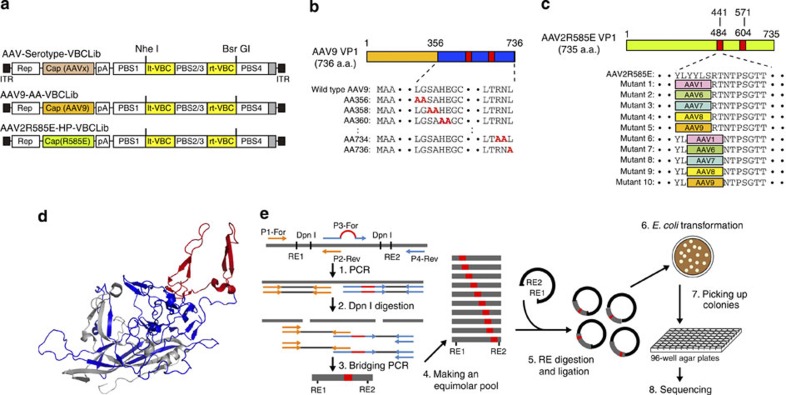
DNA-barcoded AAV libraries. (**a**) Viral genome maps of AAV-Serotype-VBCLib, AAV9-AA-VBCLib, and AAV2R585E-HP-VBCLib. Each viral genome contains a pair of 12 nucleotide-long DNA barcodes (lt-VBC and rt-VBC) downstream of the AAV2 polyadenylation signal (pA). PBS1–4, PCR primer-binding sites. Nhe I and Bsr GI indicate the restriction enzyme recognition sites used for DNA barcode cloning. (**b**) Double alanine (AA) mutagenesis of the C-terminal half of the AAV9 VP1 capsid protein from amino-acid positions 356 to 736. AAxxx’s are the names of AA mutants. (**c**) HP scanning mutagenesis of the AAV2R585E capsid. We moved HPs derived from AAV1, 6, 7, 8 and 9 on the AAV2R585E capsid at a two amino-acid interval within the regions of interest. (**d**) Structure of the AAV capsid VP protein indicating the region we investigated in this study. The blue region was examined for AAV9 mutants, and the red region was examined for both AAV9 mutants and AAV2R585E mutants. The same colours are also used to indicate the regions in **b** and **c**. The AAV9 capsid structure is presented as a representative of all the serotypes used in this study. (**e**) A schematic representation of the high-throughput site-directed capsid mutagenesis procedure used. We applied a bridging PCR technique using an artificially created codon-modified DNA as a template, which had at least two Dpn I sites between the P1-For and P4-Rev common PCR primer-binding sites. P2-Rev and P3-For are a mutation-specific set of PCR primers. We pooled up to 50 PCR amplicons at an equimolar ratio, created a mutant library and sent transformed bacteria in a 96-well format to DNA sequencing service for screening.

**Figure 2:**
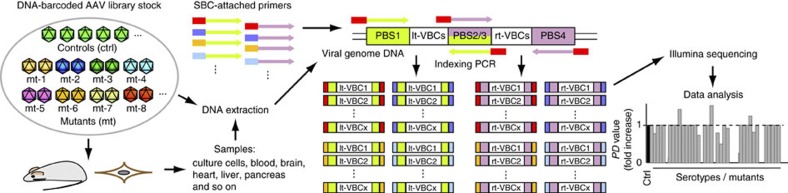
Procedure for the AAV Barcode-Seq analysis. We applied DNA-barcoded AAV library stocks to tissue culture cells for receptor binding and *in vitro* transduction assays. We injected them into mice to investigate blood clearance rate, tissue tropism, reactivity to neutralizing antibodies and anti-AAV neutralizing antibody epitope mapping. We then collected various samples, extracted DNA, PCR-amplified AAV clone-specific Virus Bar Codes (VBCs) using Sample-specific Bar Code (SBC)-attached PCR primers. We mixed all the VBC–PCR amplicons in a pool, and sequenced them on the Illumina platform. We then converted raw sequence read number data to PD values by a computational algorithm.

The aim of this study was to establish AAV Barcode-Seq as a novel method that would allow us to characterize the biological phenotypes of many AAV strains in an unprecedented high-throughput manner, and demonstrate its successful application to AAV research. To accomplish this aim, we generated the following seven DNA-barcoded AAV serotype and capsid mutant virus libraries (Table 1 and Supplementary Table 1). Each serotype or mutant viral clone in the libraries carried the wild-type AAV2 *rep* gene, an AAV *cap* gene derived from various serotypes or mutants, and a pair (pr) of left (lt) and right (rt) viral clone-specific 12-nucleotide long DNA barcodes (Virus Bar Code or VBC) (Fig. 1a). AAV-Serotype-VBCLib contained nine AAV serotypes plus two AAV chimeric mutants; AAV9-AA-VBCLib’s covered a total of 191 double alanine (AA) scanning mutants that spanned the entire region of the C-terminal half of the AAV9 VP1 capsid protein (Fig. 1b,d), and AAV2R585E-HP-VBCLib’s contained 125 AAV2R585E-derived hexapeptide (HP) scanning mutants with AAV2-derived HPs being replaced with those derived from AAV1, 6, 7, 8 and 9 capsids (Fig. 1c,d). These 125 HP mutants covered the entire region from amino-acid positions 441–484 and from 571–604. Each DNA-barcoded AAV capsid mutant library contained 15–18 clones each of AAV9 and AAV2R585E as internal reference controls in addition to 2 or 3 clones per mutant (Table 1). AAV9 AA scanning primarily focused on loss-of-function phenotypes, while heparin-binding-deficient AAV2R585E HP scanning primarily focused on gain-of-function phenotypes. To construct a large number of mutants, we established a method for high-throughput site-directed capsid mutagenesis (Fig. 1e). With these libraries, we followed the AAV Barcode-Seq experimental procedure depicted in Figure 2 to determine Phenotypic Difference (PD) values of each serotype or mutant. PD indicates ‘fold change’ of a phenotype compared with that of the reference controls.

### Validation of AAV Barcode-Seq

We validated the Barcode-Seq through analysis of errors. Over a dynamic range of 10^4^, the relative abundance of DNA templates and Illumina sequencing read numbers in a sample were positively correlated with Pearson’s correlation coefficients of 0.93 (lt-VBCs) and 0.97 (rt-VBCs) on average (Supplementary Fig. 1a). An undersampling simulation study^38^, using the actual data sets we obtained, showed that when the average Illumina sequence read number per clone is ≥64, the average coefficients of variation of the data obtained by technically replicated experiments are 0.22–0.24 and 0.31–0.34 for lt- and rt-VBCs, respectively (Supplementary Fig. 1b), and Pearson’s correlation coefficients between undersampled and full-size data sets are ≥0.96 (Supplementary Fig. 1c). We determined statistical power of the analysis by a simulated experiment that used a library containing two clones per mutant and three or more reference control clones. This simulation study showed that the statistical power to detect a twofold change with *P*<0.05 (two-tailed Mann–Whitney *U*-test) is 0.78–0.87 in a duplicated experiment and 0.87–0.96 in a triplicated experiment when the average reference control read number per clone is ≥64 (Supplementary Fig. 2). The power to detect ≥4-fold changes with *P*<0.05 (two-tailed Mann–Whitney *U*-test) was found to be ~1.0 in both duplicated and triplicated experiments (Supplementary Fig. 2).

### Proof-of-principle of AAV Barcode-Seq

Here we present four examples demonstrating that AAV Barcode-Seq can reproduce data obtained by conventional approaches. First, using the tissues collected from AAV-Serotype-VBCLib-injected mice, we determined transduction efficiencies of the major AAV serotypes in various tissues by AAV Barcode-Seq (Supplementary Fig. 3). The results reproduced the phenotypes of each AAV serotype we have already known from prior studies. For example, AAV8 and 9 transduced many tissues with high efficiency^39^,^40^,^41^,^42^,^43^, while AAV3 was the least efficient serotype in mice^41^. AAV2R585E significantly lost the ability to transduce the liver^8^. Second, we injected 1 × 10^13^ vector genomes (vg) per kg of AAV-Serotype-VBCLib (*n=*3) via the tail vein as a bolus and determined the blood clearance rate of each serotype. The results again reproduced the pharmacokinetic profiles obtained in our previous study, showing that AAV9 exhibited distinctively delayed blood clearance, while AAV2 was rapidly cleared in the early phase after injection^44^ (Supplementary Fig. 4). Third, we applied AAV-Serotype-VBCLib to Chinese hamster ovary (CHO) Pro5 and Lec2 cells to determine the ability of each serotype to bind different sugar residues in glycan chains. Pro5 cells express terminal sialic acids but do not express terminal galactose, while Lec2 cells express terminal galactose but do not express terminal sialic acids in glycan chains^45^. Using these cells, we assessed efficiencies in cell surface binding and transduction by AAV Barcode-Seq. The obtained results were consistent with the established fact that terminal N-linked sialic acids are the primary receptor for AAV1, AAV5 and AAV6 (refs 46, 47) and terminal galactose is the receptor for AAV9 (refs 24, 25) (Supplementary Fig. 5). Fourth, by injecting AAV-Serotype-VBCLib into mice pre-immunized with AAV9, we could demonstrate that anti-AAV9 neutralizing antibody crossreacts with AAV8 and AAVrh10 but does not neutralize AAV1, 2, 3, 5, 6 and 7 at an appreciable level, which is in keeping with the observation reported in the literature^48^ (Supplementary Fig. 6). These observations establish proof-of-principle of AAV Barcode-Seq.

### AAV9 capsid amino acids important for virion formation

**Figure 3:**
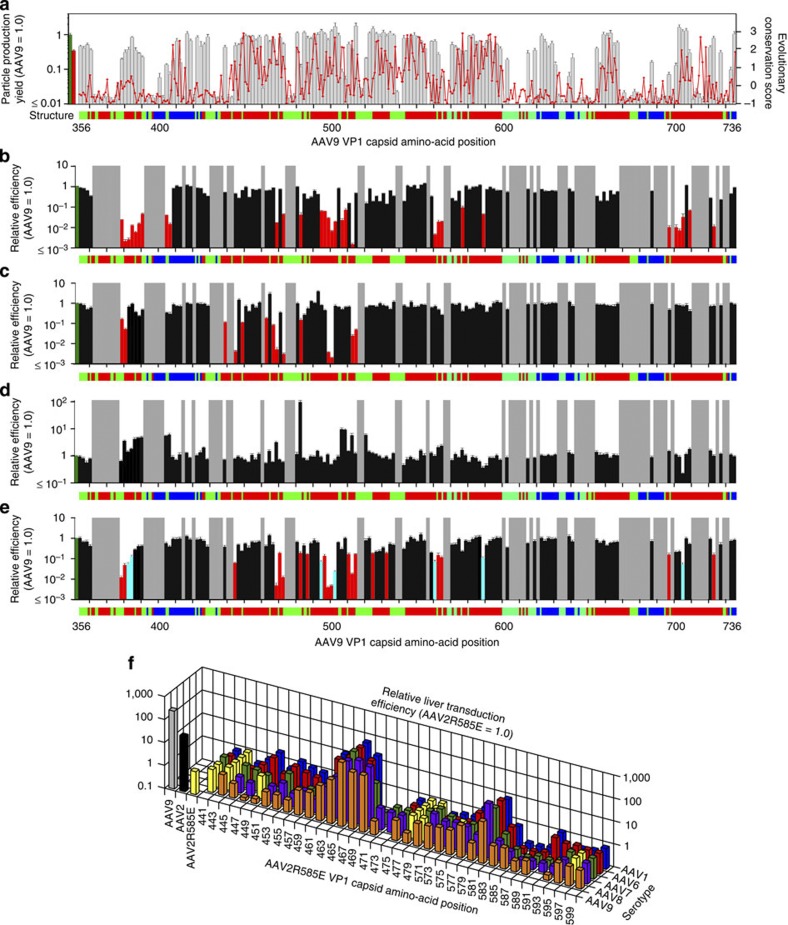
Results of the AAV Barcode-Seq analysis. (**a**) Mapping of the AAV9 capsid amino acids important for viral particle formation. Grey bars indicate viral particle production yield in HEK293 cells determined by AAV Barcode-Seq. Evolutionary conservation scores determined by the ConSurf analysis are shown with a red line. Highly conserved amino acids show low scores. Topological locations of each amino acid are shown as a horizontal colour bar. The red, blue and green regions indicate outer surface-exposed, inner surface-exposed and buried amino acids, respectively. The far left green and red bars represent the wild-type AAV9 and AAV2R585E, respectively. (**b**) Liver transduction efficiency of each AAV9 AA mutant. The mutants shown with red bars are liver-detargeting mutants (>90% reduction of liver transduction efficiency). (**c**,**d**) Results of the cell surface binding assay of the AAV9 mutants using Lec2 (**c**) and Pro5 (**d**) cells. The mutants shown with red bars in **c** are the 14 mutants exhibiting a >80% loss of the binding ability compared with the wild type. These 14 mutants were L380A/T381A, L382A/N383A, I440A/D441A, Y446A/L447A, T450A/I451A, V465A, P468A/S469A, N470A/M471A, Q474A/G475A, Y484A/R485A, E500A/F501A, W503A, R514A/N515A and S516/L517A. (**e**) Transduction efficiency of the AAV9 mutants in Lec2 cells. The mutants shown with red or cyan bars are those exhibiting a >80% loss of transduction efficiency compared with the wild type. The mutants with cyan bars are the seven mutants that showed impaired postattachment viral processing deduced by a >80% reduction of the transduction-to-binding ratios. These seven mutants were D384A/G385A, S386A/Q387A, N496A/N497A, P504A/G505A, N562A/E563A, Q590A and Y706A/K707A. In **b**–**e**, the grey regions indicate the AAV9 mutants that exhibited a >95% decrease in the viral particle formation compared with the wild type and therefore are devoid of functional phenotype data; the leftmost green bars represent the wild-type AAV9. (**f**) Liver transduction efficiency of each AAV2R585E HP mutant. Note that yellow bars are all AAV2R585E because HP replacement in them does not change their amino-acid sequence. In all the panels, error bars represent s.e.m. The number of replicates is detailed in Supplementary Data.

We then applied AAV Barcode-Seq to AAV mutants. First, we produced 382 viral clones representing 191 AAV9 AA mutants and 15 each of AAV9 and AAV2R585E reference control clones in separate culture dishes by DNA transfection, pooled an equal amount of crude cell lysate obtained from each culture dish and made three AAV9-AA-VBCLib libraries as described in Table 1. We extracted viral genome DNA from DNase I-resistant viral particles in each library, which was then subjected to the Barcode-Seq analysis. There were a total of 72 AA mutants that showed >95% reduction of intact viral particle formation compared with the wild type (Fig. 3a). As expected, a strong correlation was found between the tolerability to amino-acid substitution, the degree of evolutionary conservation and topological locations. Forty-four of the 72 mutants that could not tolerate an AA mutation had changes of amino acids buried inside the virion shell, while 28 of the 119 capsid-forming mutants had such changes (*χ*^2^ test; *P*=0.0002). Importantly, this analysis could identify amino acids that potentially have functional roles. D384, G385, I560, T561, N562, E563, E564, E565, N704 and Y705 are amino acids that are conserved and exposed on the depressed surface at the twofold symmetry axis of the capsid but tolerated mutations, indicating their functional role. Subsequent studies revealed that the mutations involving these amino acids resulted in a phenotype exhibiting impaired transduction.

### Capsid amino acids responsible for liver transduction

**Figure 4:**
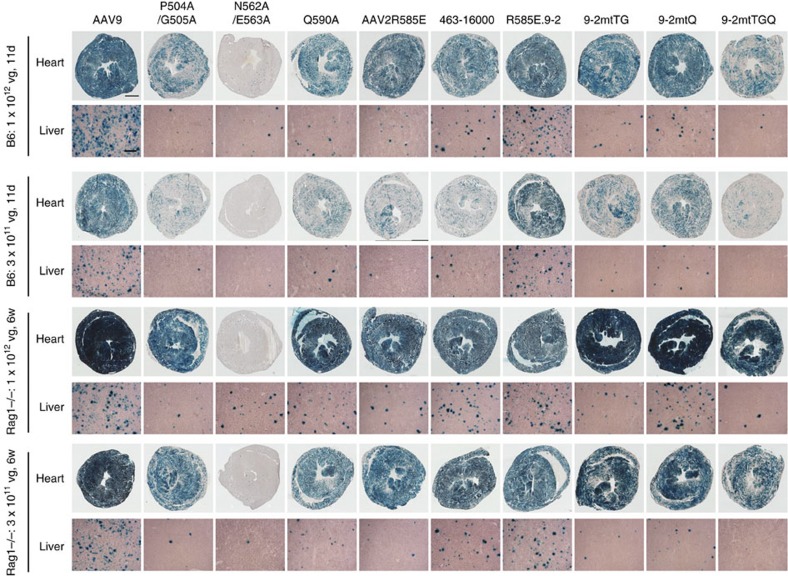
Liver and heart transduction with AAV9 and AAV2R585E mutants. We injected C57BL/6 wild type or Rag−/− mice with AAV-CMV-lacZ vector packaged with various AAV capsids at a dose of 3 × 10^11^ or 1 × 10^12^ vg per mouse intravenously (*n=*3 per group). We harvested tissues 11 days (wild type) or 6 weeks (Rag1−/−) post injection and performed X-Gal staining to determine transduction efficiency. Tissue sections were counterstained with light hematoxylin. This experiment was performed once. Scale bars, 1 mm for the heart and 200 μm for the liver.

Second, we injected C57BL/6 mice intravenously with AAV9-AA-VBCLib or AAV2R585E-HP-VBCLib at a dose of 1 × 10^12^ vg per mouse (*n=*3 per library). We harvested 12 major tissues 6 weeks post injection and subjected them to the AAV Barcode-Seq analysis, which revealed that 31 AAV9 AA mutants exhibited >10-fold reduction in liver transduction (Fig. 3b). A hierarchical clustering analysis (complete linkage method) on the *in vivo* transduction profiles in all the 12 tissues revealed that they can be grouped into two phenotypically distinct groups, that is, 9 mutants that mainly detarget the liver (Liver-Detargeting (LD) mutants) and 22 mutants showing impaired transduction not only in the liver but also in many nonhepatic tissues (Globally Detargeting (GD) mutants) (Supplementary Fig. 7). To validate these observations, we produced AAV-CMV-lacZ vector^44^ encapsidated with the following capsids: P504A/G505A (LD mutant), N562A/E563A (GD mutant) and Q590A (LD mutant). We injected them into C57BL/6 wild type or Rag1−/− mice intravenously at 3 × 10^11^ or 1 × 10^12^ vg per mouse. The results obtained by X-Gal staining, qPCR and Southern blot analyses on the tissues harvested 11 days (wild-type mice) and 6 weeks (Rag1−/− mice) post injection corroborated the AAV Barcode-Seq results (Fig. 4, Supplementary Tables 2 and 3). In particular, P504A/G505A mutant transduced the heart, kidney and brain efficiently at 92%, 318% and 60% of the wild type AAV9’s levels, respectively, with a >200-fold decrease in the liver transduction determined by vector genome copy numbers (Supplementary Table 2). In the HP scanning experiment, replacement of AAV2R585E capsid amino-acid residues 461–474 with any of the HPs derived from AAV1, 6, 7, 8 and 9 resulted in enhanced liver transduction by 3–68-fold (Fig. 3f) in all but one of the 17 virion-forming AAV2R585E-derived mutants. We validated this enhancement by injecting mice with the AAV-CMV-lacZ vector packaged with the AAV2R585E mutant 463–16000 (Fig. 4, Supplementary Tables 2 and 3). We also observed a more than sixfold increase in liver transduction when the AAV2R585E 581-586 HP was replaced with any of those derived from the other five serotypes (Fig. 3f). These observations clearly delineate the AAV1, 6, 7, 8 and 9 capsid amino-acid residues important for hepatic transduction.

### AAV9 capsid amino acids responsible for galactose binding

Third, we applied AAV9-AA-VBCLib to CHO Pro5 and Lec2 cells and investigated the ability to bind and transduce these two cell lines by AAV barcode-Seq. We found that 14 AA mutants covering 26 residues exhibited a >80% decrease in the binding to Lec2 cells (Fig. 3c), while they retained >50% ability to bind to Pro5 cells (Fig. 3d). This strongly indicates that these 26 amino acids are directly or indirectly responsible for galactose binding. Supporting this notion, 3 of the 26 residues have recently been found to constitute the galactose binding domain^9^. Topologically, they cluster in the pocket between the side walls of the threefold protrusions (Supplementary Fig. 8). All the HP scanning AAV2R585E mutants exhibited Lec2 cell binding >466-fold less than the wild-type AAV9. We also identified seven mutants showing a significantly reduced transduction-to-binding ratio compared with the wild type (Fig. 3e). The amino acids substituted in these mutants are therefore important for postattachment viral processing.

### Capsid amino acids responsible for persistence in the blood

Fourth, we applied AAV Barcode-Seq to a pharmacokinetic study of AAV mutants injected intravenously into mice. We infused 1 × 10^13^ vg per kg of each of AAV mutant libraries into mice and determined relative blood concentrations by AAV Barcode-Seq over a period of 72 h (*n=*2 per library) (Supplementary Fig. 9). At 72 h post injection, 20 AAV9 AA mutants lost persistence in the blood compared with the wild-type AAV9, showing <10% levels of the blood concentration of AAV9 (Loss of Persistence (LP) phenotype) and 13 AAV2R585E HP mutants showed 2–13-fold higher blood concentrations than AAV2R585E at 72 h post injection (Delayed Clearance (DC) phenotype). These amino-acid residues responsible for persistent circulation in the bloodstream are surface-exposed and primarily overlap with those responsible for the altered *in vivo* transduction phenotypes. Eighteen of the 20 (90%) AAV9 mutants showing an LP pharmacokinetic phenotype were LD or GD mutants (Fisher’s exact test; *P*=3 × 10^−11^; Supplementary Table 4), and 12 of the 13 (92%) AAV2R585E mutants with a DC pharmacokinetic phenotype exhibited 7–62-fold enhancement of liver transduction (Fisher’s exact test; *P*=5 × 10^−10^; Supplementary Table 5). These observations indicate that a majority of the surface-exposed amino acids that play a role in viral clearance have a dual functional role in both pharmacokinetics and *in vivo* transduction, although the amino acids important for *in vivo* transduction are not necessarily involved in the clearance (Supplementary Tables 4 and 5). One mutant, AAV9Y484A/R485A, was found to bind to various types of cells with a dramatically increased efficiency (a prominent bar in Fig. 3d) and was cleared at a strikingly accelerated rate (Supplementary Fig. 9a). The rapid blood clearance of this particular mutant could be interpreted by sequestration of viral particles from the bloodstream due to the increased cell attachment; however, the underlying mechanism for this gain-of-function phenotype remains uncharacterized.

### Anti-AAV1 and AAV9-neutralizing antibody epitope mapping

Fifth, we applied AAV Barcode-Seq to map anti-AAV1 and AAV9 capsid-neutralizing antibody epitopes. To this end, we immunized C57BL/6 mice by intravenous injection of AAV1- or AAV9-CMV-lacZ. Three weeks later, we infused AAV2R585E-HP-VBCLib into the immunized mice at a dose of 1 × 10^13^ vg per kg (*n=*3 per library) and determined viral concentrations in the blood over 1 hour by AAV Barcode-Seq. We expected that only AAV2R585E mutants with an HP containing an antibody epitope would be neutralized, and therefore would be cleared faster than other mutants in the same immunized animal or faster than the same mutant in naive animals. By taking this approach, we successfully identified 452-QSGSAQ-457 and 453-GSGQN-457 as one of the epitopes for anti-AAV1 and AAV9-neutralizing antibodies developed by viral immunization of mice, respectively (Supplementary Fig. 10). Both of the epitopes reside on the highest peak of the threefold capsid protrusions. Many neutralizing antibody epitopes are conformational, as opposed to linear. In this regard, the AAV HP scanning approach displays peptides in the context of appropriately juxtaposed regions from different parts of the sequence in a more native-like quaternary structure of viral capsid and therefore may provide a more ideal platform for the identification of epitopes than linear peptide arrays. As for anti-AAV9-neutralizing antibody epitopes, our study has indicated that there is another epitope(s) in the amino (N)-terminal half of the AAV9 capsid because AAV1.9-1 (an AAV1 and AAV9 hybrid capsid consisted of the N-terminal half of the AAV9 capsid and the C-terminal half of the AAV1 capsid)^44^ was neutralized with anti-AAV9 antibody (Supplementary Fig. 6i).

### Design of a galactose-binding motif in the AAV2 capsid

**Figure 5:**
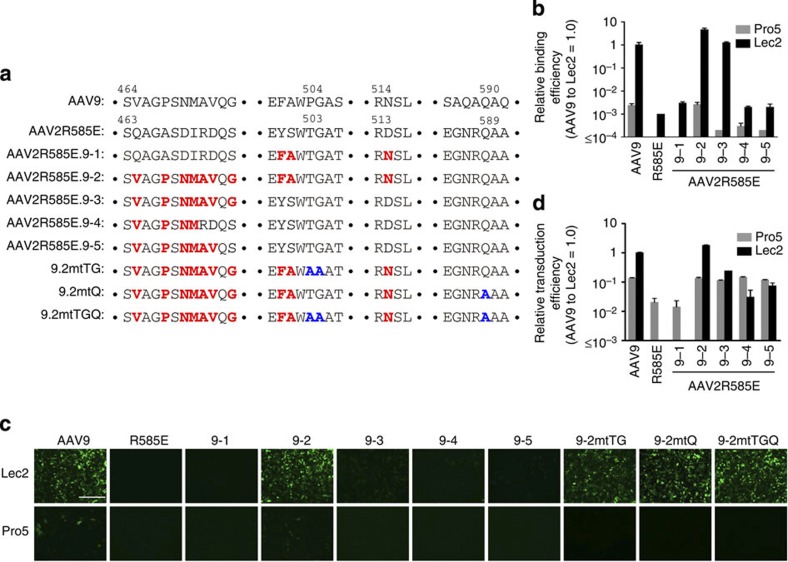
Characterization of the AAV2R585E mutants carrying amino acids responsible for galactose binding. (**a**) Capsid amino-acid sequences of a series of the AAV2R585E-derived mutants. (**b**) Cell surface binding of the AAV2R585E mutants. We produced dsAAV-CMV-GFP vectors packaged in each AAV2R585E mutant capsid. We exposed Pro5 or Lec2 cells to these mutant vectors at an MOI of 10^5^, at 4 °C for 1 h, and determined the quantity of cell surface-bound AAV particles by qPCR of the viral genome (*n=*3). (**c**,**d**) We applied the AAV2R585E mutant GFP vectors to Pro5 or Lec2 cells at an MOI of 10^6^, and determined transduction efficiency 48 h after infection by fluorescent microscopy (**c**) and flow cytometry (**d**) (*n=*3). In the bar graphs, error bars represent s.e.m. This experiment was performed once. Scale bar, 400 μm.

Now that we had identified the 26 amino acids responsible for galactose binding, we exploited this knowledge to create AAV2 capsids that bind to galactose with minimal amino-acid changes. AAV2 does not bind to galactose; therefore, successful creation of galactose-binding AAV2 mutants based on the knowledge obtained from the AAV Barcode-Seq data would provide a proof of the practical utility of the AAV Barcode-Seq analysis. To create such mutants, we selected 10 residues based on the AAV Barcode-Seq data and the information about the evolutionary conservation and topological locations of the identified amino acids. In brief, we assumed that the amino acids critical for galactose binding are those that are evolutionarily variable, surface exposed and form a cluster on the surface of the capsid. To assess the evolutionary conservation of the AAV capsid amino acids, we compared the capsid amino-acid sequences between AAV1, 2, 3, 6, 7, 8 and 9, which represent each of all the six AAV clades^48^. A sequence alignment analysis revealed that, among the 26 amino acids, the amino acids except for the following 12 amino acids, I451, V465, P468, S469, N470, M471, G475, Y484, E500, F501, N515 and L517, are completely conserved in the AAV1, 2, 3, 6, 7, 8 and 9 capsids. Among these 12 amino acids, I451, V465, P468, S469, N470, E500, F501 and N515 are surface-exposed. These amino acids cluster in the pocket that has recently been reported as the site where AAV9 binds galactose^9^. AAV9 S469 is shared with AAV2; L517 is well conserved between different serotypes and Y484 is not located in the pocket and well conserved. Therefore, we did not select these three amino acids. As for AAV9 I451, the partner of the double alanine mutation, T450, is completely conserved. For this reason, we opted not to introduce a mutation at AAV2 N449 corresponding to AAV9 I451. Consequently, we introduced the following 10 amino-acid substitutions to the AAV2 capsid to create a new galactose-binding motif in the AAV2R585E capsid: Q464V/A467P/D469N/I470M/R471A/D472V/S474G/Y500F/S501A/D514N (Fig. 5a). R471A/D472V was included because the AAV9AA472 with a V473A mutation within the vicinity of this amino-acid cluster also showed impairment of Lec2 cell binding (PD=0.36).

We then replaced some or all of these 10 residues spanning from 464 to 514 in the AAV2R585E capsid with those of AAV9 and produced AAV2R585E.9-1, 9-2, 9-3, 9-4 and 9-5 vectors expressing GFP or lacZ driven by the CMV promoter (Fig. 5a). 9-2 contained all the 10 residue substitutions while 9-1 and 9-3 contained only the right or left arm of the motif, respectively. 9-4 and 9-5 were among the AAV2R585E HP mutants. We infected Pro5 and Lec2 cells with all the five mutants together with controls and injected mice with 9-2 in the same manner as described above. This experiment revealed that 9-2 has biological properties that are almost the same as those of the wild-type AAV9 both *in vitro* (Fig. 5b–d) and *in vivo* (Fig. 4, Supplementary Tables 2 and 3). 9-2 bound and transduced Lec2 cells and exhibited a high liver and heart transduction efficiency. The other four mutants showed some reduction compared with the wild-type AAV9. This experiment also revealed that AAV9 F501, A502 and/or N515 are important not only for galactose binding but also for postattachment viral processing because 9-3 bound to Lec2 cells at a level comparable to that of 9-2 and AAV9 but showed significantly attenuated Lec2 transduction. Shen *et al.*^49^ have recently created an AAV2 capsid that binds to terminal galactose based on our AAV Barcode-Seq data reported earlier in a scientific meeting, which independently validates the AAV Barcode-Seq approach and data.

### Design of liver-detargeting mutants

P504A/G505A and Q590A mutations in AAV9 capsid led to a liver-detargeting phenotype that preserved the ability to mediate efficient cardiac transduction in mice. T503/G504 and Q589 in AAV2 are residues corresponding to P504/G505 and Q590 in AAV9. To investigate whether introduction of alanine mutations to these residues in the AAV2R585E.9-2 capsid also attenuates the ability to mediate liver transduction, we created three AAV2R585E.9-2 capsid mutants carrying T503A/G504A (mtTG), Q589A (mtQ) or T503A/G504A/Q589A (mtTGQ) (Fig. 5a) and packaged the lacZ- or GFP-expressing AAV viral genome into them. We tested these AAV mutant vectors *in vitro* and *in vivo* in the same manner as described above. We did not observe any phenotypic changes *in vitro* (Fig. 5c). However, in mice, we found that mtTG substantially attenuated liver transduction and mtTGQ nearly completely abolished liver transduction, while both of them retained the capability of transducing the heart (Fig. 4, Supplementary Tables 2 and 3). Although Q589A had only an ancillary role in the context of AAV2R585E.9-2, this study also demonstrated that it is feasible to design new AAV mutants with directed phenotypes based on the knowledge obtained from the AAV Barcode-Seq data.

### High-resolution functional maps of the AAV9 capsid

**Figure 6:**
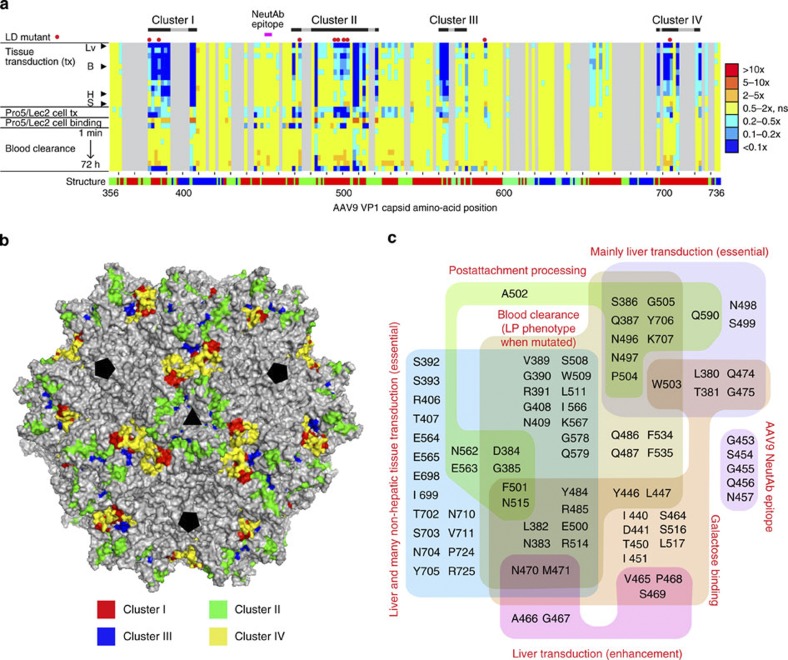
Functional maps of the C-terminal half of the AAV9 capsid amino acids. (**a**) A 2D heat map showing correlations between positions of AA mutations and phenotypic changes. Degrees of phenotypic changes caused by AA mutations are categorized into seven groups as indicated to the right. Functionally important amino acids form four clusters (Clusters I–IV) that are partially discontinuous by the presence of the amino acids responsible for the structural integrity shown as grey regions. The following is the orders of the samples in the heatmap (from the top to the bottom): liver (Lv), skeletal muscle, dorsal skin, intestine, brain (B), visceral fat, lung, pancreas, testis, heart (H), kidney and spleen (S) for the tissue samples; Pro5 transduction, Lec2 transduction, Pro5 binding and Lec2 binding for the *in vitro* samples and 1 min, 10 min, 30 min, 1 h, 4 h, 8 h, 24 h and 72 h time points for the blood samples. No significant phenotypic changes (ns, indicated with yellow) are those showing a greater than fourfold increase or decrease in a PD value with no statistical significance (two-tailed Mann–Whitney *U*-test, *P*≥0.05). (**b**) A 3D map on the full AAV9 capsid atomic model showing topological locations of the functionally important amino acids in Clusters I–IV. A triangle and three pentagons indicate three- and fivefold symmetry axes, respectively. Twofold symmetry axes are in the centre of two adjacent fivefold symmetry axes. The image was generated by PyMOL. (**c**) A Venn diagram showing correlation between AAV9 capsid amino-acid residues and various AAV9 phenotypes. The AAV9 neutralizing antibody epitope, the amino acids responsible for enhanced liver transduction, and 3 of the 16 amino acids important for postattachment processing, F501, A502 and N515, were identified through the AAV2R585E mutants. All other amino acids and their phenotypes were identified by the AA mutagenesis study of the AAV9 capsid. Owing to the nature of the AA mutagenesis approach, it remains unknown which one of the two amino acids mutated to alanine is responsible for the phenotypes or whether both of the two amino acids are responsible for the phenotypes.

Finally, with all the data being combined, we were able to draw high-resolution functional maps of the amino-acid residues in the C-terminal half of the AAV9 capsid. By two-dimensional (2D) heat mapping, one can readily appreciate that functionally important amino acids form four clusters (Clusters I–IV, Fig. 6a). Cluster II contains the most amino-acid residues causing the LD phenotype when mutated. On the other hand, functionally important amino acids in Clusters I, III and IV are dominated by those that play a more fundamental role in any transduction events. In keeping with this functional distinction between the clusters, a three-dimensional (3D) mapping of Clusters I–IV reveals that Cluster II is on the side walls of the threefold capsid protrusions and topologically distinct from the Clusters I, III and IV, which mainly reside in the twofold axis valley on the capsid (Fig. 6b). By displaying the data in a Venn diagram, it is revealed that many of the functionally important amino-acid residues, particularly those in Cluster II, play a multifunctional role (Fig. 6c). Although detailed investigation and interpretation of each of many amino acid–phenotype correlations we have identified is out of the scope of this article, all the data presented here demonstrate that the Barcode-Seq approach combined with DNA barcode-tagged mutagenesis offers a novel powerful means to study multifunctional viral capsid proteins and seek clues in designing novel viral vectors for gene therapy.

## Discussion

We present here a novel NGS-based methodology to comprehensively characterize various aspects of biological phenotypes of many AAV strains in a high-throughput manner. Unlike the commonly used AAV capsid directed evolution methods to screen random peptide display libraries or shuffled capsid libraries^34^,^35^,^36^,^37^, AAV Barcode-Seq *per se* is not aimed at identifying improved AAV capsids with desired phenotypes. Rather, AAV Barcode-Seq is a method that has enormous power to collect a large data set on capsid amino-acid sequence–phenotype correlation in a short period of time, using only a small number of replicates. We show that, when this new method is applied to capsid mutant libraries, comprehensive functional dissection of multifunctional viral capsid proteins becomes possible. Such functional mapping of viral capsids, which was not possible by conventional methodologies, significantly furthers our understanding of viral capsid biology and unambiguously identifies functional amino-acid residues that would provide important targets for protein engineering directed at altered functions. Similar NGS-based approaches have recently been reported in other experimental settings^38^,^50^,^51^,^52^. However, to the best of our knowledge, this is the first demonstration that an NGS-based approach enables us to draw a comprehensive high-resolution functional map of a multifunctional protein such as the AAV capsid protein we studied in this report.

AAV Barcode-Seq should not be mistaken for a complicated and labour-intensive approach. Although the validation studies reported here required substantial time and efforts, Barcode-Seq *per se* is a straightforward and relatively effortless procedure that involves only the following routine molecular biology techniques and basic bioinformatics tasks: DNA extraction from samples, DNA-PCR, Illumina sequencing of PCR products and data analysis. Illumina sequencing can be done at NGS resources at a steadily decreasing cost, and NGS data analysis has now become a standard bioinformatic task in many molecular biology laboratories as well as bioinformatics laboratories. In addition, the labour required for one DNA-barcoded AAV library production and purification is not substantially more than that for one conventional AAV vector preparation once all plasmid DNAs for AAV production have been prepared. For example, we will be able to compare relative transduction efficiencies of 10 different laboratory-engineered AAV strains of interest in major organs in AAV-injected animals using only three animals and one DNA-barcoded AAV library if we take the AAV Barcode-Seq approach. In contrast, for a side-by-side comparison, conventional approaches would require at least 30 animals (*n=*3 or more per AAV strain) and 10 AAV preparations that are individually prepared and purified on a scale comparable to that of the DNA-barcoded AAV library.

We used the AAV *rep–cap* genome for this proof-of-concept study; however, AAV viral genomes tagged with DNA barcodes can be any type of DNA devoid of the *rep* and *cap* genes. The core DNA sequence essential for our Barcode-Seq approach is the 96 nucleotide-long DNA barcode cassette composed of a pair of random 12 nucleotides, three PCR primer-binding sites and two restriction enzyme recognition sites for Nhe I and Bsr GI (Fig. 1a). This DNA barcode cassette has been thoroughly validated for the Barcode-Seq analysis and in theory should function in any context of DNA sequence. The novel approach reported here will therefore readily be evolved into a more universal system by incorporating the DNA barcode cassette into a standard recombinant AAV viral genome devoid of the *rep* and *cap* genes and supplying these viral genes *in trans* as an AAV helper plasmid when DNA-barcoded AAV libraries are produced. Thus, tissue tropism, transduction efficiencies and any aspects of biological properties of a panel of many AAV capsid mutants of interest expressing a marker gene or a therapeutic gene can readily be compared side-by-side by AAV Barcode-Seq using DNA-barcoded AAV libraries produced by the standard three plasmid transfection method^53^,^54^ without a need of constructing the AAV *rep–cap* genomes with DNA barcodes. A potential caveat of AAV Barcode-Seq is that the quantity of AAV vector genome DNA in cells may not necessarily correlate with the level of transgene expression. This caveat, however, will be addressed by co-transcribing DNA barcodes into RNA. Such an RNA barcode-based system will allow relative quantification of AAV vector-mediated transgene expression levels from many AAV mutants in a limited number of replicates. Thus, the AAV Barcode-Seq approach and more broadly high-throughput phenotypic characterization of barcode-tagged viruses by NGS in general have a potential to expand their utility, immediately and in the future, beyond the scope of the study reported here.

An important question in viral vector research is how a spectrum of biological properties of vectors is determined by viral components. In this regard, the large data set we generated and the AAV capsid functional map we presented here not only reinforce previous observations but also provide new insights into the AAV capsid biology. For example, one can now appreciate that liver transduction of AAV9 is governed by two mechanisms: one requiring galactose binding and another that utilizes an independent pathway(s) (Fig. 6c). This in part supports the previous notion that a decrease in galactose binding avidity results in a liver-detargeting phenotype with systemic transduction^6^. However, this notion is not entirely correct because our study shows the presence of galactose binding-independent liver transduction pathways involving several amino acids such as P504, G505 and Q590. The observations from galactose binding-deficient AAV9 mutants E500A/F501A and W503A and a galactose binding-proficient and transduction-impaired mutant AAV2R585E.9-3 (or AAV2R585E.9-2 F500Y/A501S/N514D) indicate the potential presence of an amino-acid residue(s) in the vicinity of E500-W503 in the AAV9 capsid that is not only responsible for galactose binding but also involved in postattachment viral processing. Such a dual role of amino-acid residues provides an alternative interpretation for the liver-detargeting nature of galactose binding-deficient mutants, such as AAV9W503R reported by Shen *et al.*^6^ and AAV9W503A described in this study. That is, impaired postattachment viral processing and decreased galactose binding can coincidentally result from a mutation, while the mere ablation of galactose binding does not necessarily result in a liver-detargeted phenotype.

Another new insight is that AAV9-mediated liver transduction likely requires an additional viral processing step that is not required for transduction in nonhepatic tissues. This insight comes from the following observation: AAV9 mutants that showed a >90% decrease (dark blue in Fig. 6a) in transduction in any of the nonhepatic tissues also exclusively exhibited a >90% decrease in liver transduction (odds>11), while impaired liver transduction did not necessarily accompany significantly attenuated transduction in multiple nonhepatic tissues, as observed in the LD mutants (odds=0.24–0.81). The straightforward interpretation of this observation is that the liver poses an additional hurdle to AAV9 to achieve transduction and the AAV9 capsid possesses amino-acid residues that are functionally required for overcoming this hurdle but are not required for overcoming transduction barriers in nonhepatic tissues. Although the nature of this liver-specific transduction barrier has yet to be elucidated, our study has indicated that it could be a postattachment barrier because more than a half of the amino acids associated with the LD phenotype also play a role in postattachment viral processing (Fig. 6c).

In summary, we have successfully established a new method to collect a large data set on amino-acid sequence-viral capsid phenotype correlation in a high-throughput manner and used it to draw a high-resolution functional map of the AAV capsid protein. A complete AAV Barcode-Seq data set on phenotypes of all the AAV serotypes and mutants we analysed is provided as Supplementary Data 1–3. As more data accumulate and are explored by further data mining, we will be able to understand AAV virus and vector biology in much greater detail. AAV Barcode-Seq, when combined with other complementing methodologies, will ultimately allow us to design and create novel AAV vectors endowed with the most desirable biological properties for each clinical application. Importantly, this approach can be readily adapted to studies involving animals with more translational relevance, such as nonhuman primates.

## Methods

### Cell culture conditions and experiments

Human embryonic kidney (HEK) 293 cells, AAV293, were purchased from Stratagene. CHO Pro5 and Lec2 cells were gifted by A. Asokan (University of North Carolina, Chapel Hill). HEK293 cells were grown in Dulbecco’s Modified Eagle’s Medium (DMEM, Lonza) and CHO Pro5 and Lec2 cells were maintained in Alpha-Minimum Essential Medium (Alpha-MEM, Sigma-Aldrich). The media were supplemented with 10% fetal bovine serum (FBS), L-glutamine and penicillin–streptomycin. Frozen cell stocks were created without further authentication from the original vials we received before use for experiments. These cell lines have been tested for mycoplasma contamination using a mycoplasmal 16S rDNA PCR assay and found to be negative. The virus cell surface binding and transduction assays were performed using CHO Pro5 and Lec2 cells seeded on 24- or 96-well plates 1 day before each experiment as detailed in Supplementary Methods.

### Plasmid construction

pAAV-Serotype-x-VBC-y (Serotype-x=serotype 1, serotype 2, serotype 3, …), pAAV9-SBBANN-AA-x-VBC-y (AA-x=AA356, AA358, AA360, …) and pAAV2R585E-SBBXEB-HP-x-VBC-y (HP-x =441-00700, 441-16,000, 443-00009, …) are the AAV plasmids with which we produced each AAV serotype and mutant viral clone with a clone-specific pr-VBC-y (y=1, 2, 3, …). These plasmids are all derivatives of pAAV9-SBBANN-VBCLib (accession code, KF032296) or pAAV2R585E-SBBXEB-VBCLib (accession code, KF032297). The detailed methodological information about plasmid construction and high-throughput double alanine and hexapeptide scanning mutagenesis can be found in Supplementary Methods.

### Production of DNA-barcoded AAV libraries and AAV vectors

We produced DNA-barcoded AAV libraries using an adenovirus-free plasmid transfection method and purified them by two cycles of caesium chloride ultracentrifugation^55^,^56^. We transfected HEK293 cells with 15 μg of each AAV library clone plasmid (that is, either pAAV-Serotype-x-VBC-y, pAAV9-SBBANN-AA-x-VBC-y or pAAV2R585E-SBBXEB-HP-x-VBC-y) together with 15 μg of pHelper (Stratagene) in separate 15-cm dishes by a calcium phosphate transfection method. Forty-eight hours after transfection, we harvested the cells and resuspended them in 1 ml of cell suspension buffer (50 mM Tris–HCl (pH 8.5), 2 mM MgCl_2_) in separate tubes, performed three cycles of freezing and thawing, and obtained individual crude cell lysates. We mixed 10 μl of each crude cell lysate to be contained in a library, and made a pool of crude lysates. We extracted viral genome DNA from Benzonase (MERCK KGaA)-resistant AAV particles and performed the AAV Barcode-Seq analysis as detailed elsewhere to assess the relative quantity of each viral clone in the pool. We used this information to adjust the quantity of each crude lysate to be mixed into a larger pool of the crude lysates; however, we did not make extensive adjustment due to the relative nature of the AAV Barcode-Seq analysis. We then purified this larger pool of the crude lysates by our standard AAV vector purification procedure based on two cycles of caesium chloride ultracentrifugation^55^,^56^. The resulting agents were the AAV library stocks used in the study. Each AAV viral clones were mixed into each of the seven libraries as summarized in Table 1. As for AAV9-AA-VBCLib’s, we split the AAV9 AA mutants into four AAV9-AA-VBCLib’s in such a way that 119 capsid-forming AAV9 AA mutants were analysed twice in two different libraries. We produced and purified single-stranded AAV-CMV-lacZ and double-stranded (ds) AAV-CMV-GFP vectors packaged with different serotype and mutant capsids^44^,^57^ using the standard three-plasmid transfection method followed by two cycles of caesium chloride ultracentrifugation^55^,^56^.

DNase I-resistant AAV particle titres were determined by a quantitative dot blot assay using an AAV2 *rep* gene probe. The relative viral particle production yield of each serotype or mutant AAV was determined as detailed in Supplementary Methods. As described in the main text, 72 of the 191 AA mutants did not produce AAV viral particles sufficient for the downstream analyses. Of the 125 HP scanning AAV2R585E mutants, the following 8 mutants, 459-00700, 461-00700, 463-00700, 469-16000, 471-16000, 473-00009, 473-16000 and 591-00009 showed a >95% decrease in AAV viral particle production compared with AAV2R585E. Therefore, these 72 AAV9 mutants and 8 AAV2R585E mutants were excluded from the downstream phenotypic analyses. In AAV-Serotype-VBCLib, we lost a substantial titre of AAV4 clones during the AAV library production; therefore, AAV4 was excluded from the downstream phenotypic analyses.

### Animal experiments

All the animal experiments were performed according to the guidelines for animal care at University of Pittsburgh and Oregon Health & Science University. We used 8-week-old C57BL/6 male mice and C57BL/6 Rag1−/− male mice purchased from the Jackson Laboratory. We randomly assigned each animal to each experimental group without considering body weights of animals. In the AAV Barcode-Seq studies, we injected C57BL/6 mice with AAV libraries intravenously at a dose of 1 × 10^12^ vg per mouse for the assessment tissue transduction efficiencies, and at a dose of 1 × 10^13^ vg per kg for pharmacokinetic studies^44^. To immunize C57BL/6 mice with AAV1 or AAV9, we injected mice intravenously with 1 × 10^11^ vg per mouse of AAV-CMV-lacZ vector packaged with the AAV1 or AAV9 capsid, respectively. We used *n=*2 or 3 per library in the AAV Barcode-Seq studies as detailed in Supplementary Data 1, 2 and 3. This sample size has been justified by the statistical power of the AAV Barcode-Seq analysis as shown in Supplementary Fig. 2. To validate the AAV Barcode-Seq results and investigate tissue tropism and transduction efficiencies of each mutant, we injected 8-week-old C57BL/6 male mice and C57BL/6 Rag1−/− male mice with AAV-CMV-lacZ vector packaged with AAV capsids of interest intravenously at two doses, 3 × 10^11^ and 1 × 10^12^ vg per mouse. For this validation study, we used *n=*3 per group. We sacrificed the vector-injected C57BL/6 mice and C57BL/6 Rag1−/− mice 11 days and 6 weeks post-injection, respectively, to determine transduction efficiencies in the following 12 major organs: brain, heart, lung, liver, kidney, spleen, intestine, pancreas, testis, hind limb skeletal muscle, visceral adipose tissue and dorsal skin. All the animal experiments were performed in a non-blinded fashion.

### AAV Barcode-Seq analysis

We extracted total DNA from cultured cells and tissues using Nucleospin Tissue Kit (MACHEREY-NAGEL, Duren, Germany) or by phenol–chloroform. We used 100 ng of total DNA to PCR-amplify lt- and rt-VBCs (that is, VBC-PCR). We extracted viral DNA from AAV library stocks using Wako DNA Extraction Kit (Wako Chemicals, Richmond, USA) and used viral DNA molecules equivalent to 1–2 × 10^8^ particles for VBC-PCR. For blood samples, we amplified VBCs directly from the blood without extracting DNA using the lysis and neutralization buffers that come with Extract-N-Amp Blood PCR Kit (Sigma, Saint Louis, USA). We used 0.1 μl of the whole blood for PCR. This quantity gave us PCR signals sufficient for the downstream analysis and was empirically determined. The PCR primers we used for the amplification of lt- and rt-VBCs are as follows: lt-VBC-For (FSN-SBC-ACCTACGTACTTCCGCTCAT), lt-VBC-Rev (FSN-SBC-TCCCGACATCGTATTTCCGT), rt-VBC-For (FSN-SBC-ACGGAAATACGATGTCGGGA) and rt-VBC-Rev (FSN-SBC-CTTCTCGTTGGGGTCTTTGC). Each primer had a 3–4 nucleotide-long Sample-specific Bar Code (SBC) and a 0–4 Frame-Shifting Nucleotides (FSN) at the 5′ end. We incorporated SBCs for multiplexed Illumina sequencing and FSNs for overcoming the issue of low sequence diversity of PCR products in reference image construction^58^. The PCR cycles used for the VBC-PCR were 2 min at 95 °C, 35 cycles of 15 s at 95 °C and 30 s at 68 °C, and subsequently 5 min at 68 °C. We quantified each PCR product by agarose gel electrophoresis followed by densitometry of ethidium bromide-stained DNA. We then pooled up to 96 multiplexed PCR products at an equimolar ratio and performed Illumina sequencing as detailed in Supplementary Methods. We assessed the quality of Illumina raw sequence reads by FastQC ( http://www.bioinformatics.babraham.ac.uk/projects/fastqc/). The following quality measures, that is, per base sequence quality, per sequence quality scores, per base N content and sequence length distribution, were all met in all the data sets we used in this study. We extracted the data on sequence read numbers of each lt- and rt-VBCs in each sample from Illumina fastq files. To do this, we developed an algorithm for binning sequence reads by SBC, subsequently by lt- and rt-VBCs, and implemented it in Perl at the Pittsburgh Supercomputing Center.

In the AAV Barcode-Seq analysis of a sample, we determined PD (*x*_*i*_*, y*_*k*_), which indicates a Phenotypic Difference between an AAV strain *x*_*i*_ (AAV*x*_*i*_) and the reference control strain *y*_*k*_ (AAV*y*_*k*_) contained in an AAV library, based on Illumina sequence read numbers. To determine PD (*x*_*i*_*, y*_*k*_), we defined the following functions: *RNLS_lt_VBC* (*x*_*ij*_), *RNLS_rt_VBC* (*x*_*ij*_), *RNES_lt_VBC* (*x*_*ij*_*, n*), *RNES_rt_VBC* (*x*_*ij*_*, n*), *RR_lt_VBC* (*x*_*ij*_*, n*), *RR_rt_VBC* (*x*_*ij*_*, n*), *GNR_lt_VBC* (*x*_*ij*_*, y*_*k*_*, n*), and *GNR_rt_VBC* (*x*_*ij*_*, y*_*k*_*, n*) (*i*
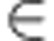
{1, 2,..., N_1_}, *j*
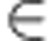
{1, 2, …, N_2_, …, N_3_}, *k*
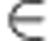
{1, 2} and *n*
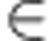
{1, 2, …, N_4_}). In *x*_*ij*_, *i* and *j* refer to AAV strains and AAV clones of the same AAV strain, respectively. *y*_*k*_ represents reference control AAV strains and takes only two values; AAV*y*_1_=AAV9 and AAV*y*_2_=AAV2R585E. In these functions, RNLS, RNES, RR, GNR and PD stand for Read Number in Library Stock, Read Number in Experimental Sample, Raw Ratio, Globally Normalized Ratio and Phenotypic Difference, respectively; *N*_1_ is the number of AAV strains contained in an AAV library; *N*_2_ is the number of AAV clones derived from the same AAV*x*_*i*_ (*i*≥3) in the library; *N*_3_ is the maximum number of AAV clones representing the same AAV strain, which is the same as the number of clones of the reference controls; *N*_4_ is the number of replicates. AAV*x*_1*j*_ and AAV*x*_2*j*_ represent the reference control AAV9 and AAV2R585E clones, respectively. For example, AAV9-AA-VBCLib-1 (Table 1) contains 15 AAV9 reference control clones, 15 AAV2R585E reference control clones and 184 AAV9 mutant clones representing 92 different AAV9 mutants (that is, 2 clones per mutant). Therefore, *N*_1_, *N*_2_, *N*_3_ and the number of *ij* combinations of this library are 94, 2, 15 and 214, respectively; AAV*x*_1*j*_ (*j*
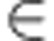
{1, 2,..., 15}) are AAV9 clones; AAV*x*_2*j*_ (*j*
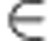
{1, 2,..., 15}) are AAV2R585E clones, and AAV*x*_*ij*_ (*i*
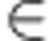
{3, 4,..., 94}, *j*
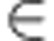
{1, 2}) are AAV9 mutant clones.

*RNLS_lt_VBC* (*x*_*ij*_) and *RNLS_rt_VBC* (*x*_*ij*_) are Illumina sequence read numbers of lt-VBC of AAV*x*_*ij*_ and those of rt-VBC of AAV*x*_*ij*_, respectively. These numbers represent an AAV library stock used in a set of replicated experiments. *RNES_lt_VBC* (*x*_*ij*_*, n*) and *RNES_rt_VBC* (*x*_*ij*_*, n*) are Illumina sequence read numbers of lt-VBC of AAV*x*_*ij*_ and those of rt-VBC of AAV*x*_*ij*_, respectively, representing a sample obtained from the replicated experimental set ‘*n*’ that uses the same AAV library stock. If an experiment is done in triplicate (experimental sets 1, 2 and 3), *n=*1, 2 or 3 is given to each experimental set. With these values, we calculated *RR_lt_VBC* (*x*_*ij*_*, n*) and *RR_rt_VBC* (*x*_*ij*_*, n*) using the following formulas (1, 2):


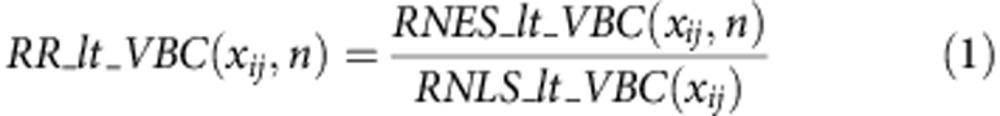



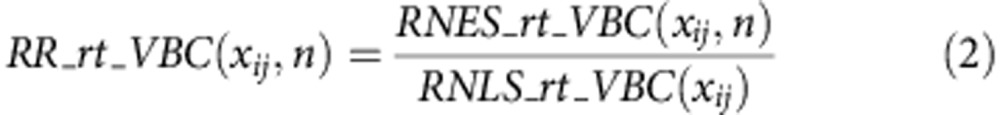


*GNR_lt_VBC* (*x*_*ij*_*, y*_*k*_*, n*) and *GNR_rt_VBC* (*x*_*ij*_*, y*_*k*_*, n*) are globally normalized *RR_lt_VBC* (*x*_*ij*_*, n*) and *RR-rt_VBC* (*x*_*ij*_*, n*), respectively, when the reference control AAV*y*_*k*_ is selected for comparison. We calculated *GNR_lt_VBC* (*x*_*ij*_*, y*_*k*_*, n*) and *GNR_rt_VBC* (*x*_*ij*_*, y*_*k*_*, n*) using the following formulas (3, 4):


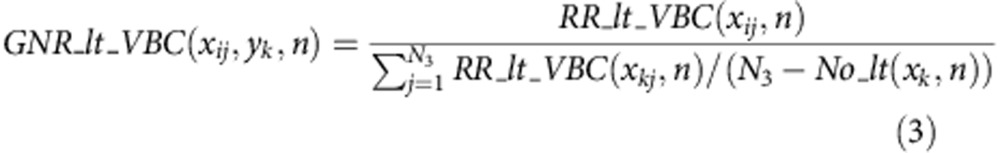



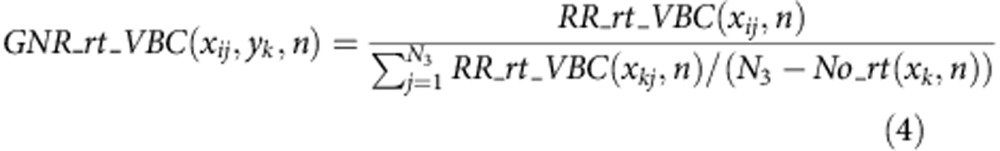


Please note that *No_lt* (*x*_*k*_*, n*) *and No_rt* (*x*_*k*_*, n*) are the number of outliers in the *RR_lt_VBC* and *RR_rt_VBC* values of reference controls (that is, AAV*x*_1_ or AAV*x*_2_) identified based on the three times the interquartile range. Therefore, when outliers were removed, the number of summation elements in the above formula was fewer by the number of outliers.

We then calculated the PD (*x*_*i*_*, y*_*k*_) (*i*≥3), which represents the average of *GNR_lt_VBC* and *GNR_rt_VBC* values, using the following formula (5):


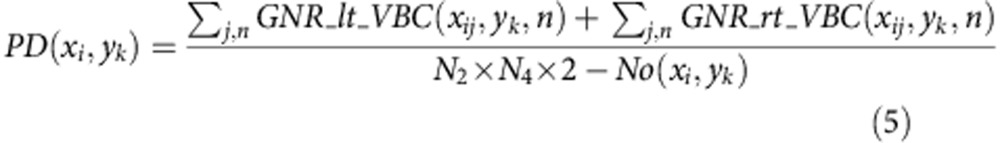


*No* (*x*_*i*_*, y*_*k*_) is the number of outliers in the *GNR_lt_VBC* and *GNR_rt_VBC* values of AAV strains besides the reference controls (AAV*x*_*i*_, *i*≥3), identified based on the three times the interquartile range. Therefore, when outliers were removed, the number of summation elements in the above formula was fewer by the number of outliers identified in each *GNR_lt_VBC* and *GNR_rt_VBC* data set. In this manner, the PD values for the reference controls (that is, PD (*x*_1_*, y*_1_) and PD (*x*_2_*, y*_2_)) always stay 1. Therefore, PD (*x*_*i*_*, y*_*k*_) provides ‘fold increase’ values of AAV*x*_*i*_ compared with the reference control AAV*y*_*k*_. In the actual experiment, we always obtained Illumina sequence read numbers from three independent sets of lt- and rt-VBC PCR amplicons from AAV library stocks, while we obtained only one set of lt- and rt-VBC PCR amplicons from samples. Therefore, we calculated *GNR_lt_VBC* (*x*_*ij*_*, y*_*k*_*, n*) and *GNR_rt_VBC* (*x*_*ij*_*, y*_*k*_*, n*) using each of the three sets of the AAV library stock data, and used the averages of the three sets of *GNR_lt_VBC* (*x*_*ij*_*, y*_*k*_*, n*) and *GNR_rt_VBC* (*x*_*ij*_*, y*_*k*_*, n*) to determine PD (*x*_*i*_*, y*_*k*_).

All the PD values were statistically assessed by two-tailed Mann–Whitney *U*-test and given a *P*-value to each mutant–phenotype combination as detailed in the ‘Statistical analysis’ subsection in the Methods. PD values showing a fourfold increase or decrease with *P*≥0.05 due to a significant degree of data dispersion were not used to assess the mutation-phenotype relationships. We set up a value that distinguishes mutants showing a substantial decrease in PD values from others in the following phenotypes to interpret the data. We took >90% decrease for liver transduction, >80% decrease for CHO cell binding and CHO cell transduction and >90% decrease for the LP pharmacokinetic property. Although a higher or lower value could be considered, these thresholds could provide meaningful interpretation of the data as shown in the Results section.

### Analyses of tissue transduction

We determined tissue transduction efficiencies in AAV-CMV-lacZ vector-injected mice by X-Gal staining, Southern blot analysis and/or qPCR. Briefly, we histologically determined transduction efficiencies in the liver by manually counting X-Gal-positive cells and negative cells and in the heart by an image analysis using MetaMorph software^44^. We determined AAV vector genome copy numbers in the liver by Southern blot analysis^44^ and those in nonhepatic tissues by qPCR. In some liver samples, vector genome copy numbers were determined by qPCR. For the qPCR assay, we mixed 100 ng of total DNA with Power SYBR Green Master Mix Reagents and PCR primers (10 pmol each per reaction) in a total volume of 25 μl and performed qPCR using Rotor-Gene Q. We amplified the CMV promoter sequence and the mouse agouti gene sequence for vector genome quantification and normalization, respectively. As for the copy number standards, we used supercoiled circular plasmids containing each of the PCR target sequences. The qPCR primer sequences are as follows: CMV-P forward (5′-TGGGAGTTTGTTTTGCACCAA-3′), CMV-P reverse (5′-CGCCTACCGCCCATTTG-3′), Mouse-agouti forward (5′-GGCGTGGTCAGTGGTTGTG-3′) and Mouse-agouti reverse (5′-TTTAGCTTCCACTAGGTTTCCTAGAAA-3′). Vector genome copy numbers were expressed as double-stranded vector genome copy numbers per diploid genomic equivalent.

### Bioinformatics and computer modelling of the AAV capsids

We collected nucleotide sequence information of 128 AAV strains from GenBank and calculated evolutionary conservation scores by ConSurf with the default parameters^58^,^59^. We obtained 3D structure coordinates of the AAV2 and AAV9 capsids from the Protein Data Bank (1lp3 and 3ux1 for AAV2 and AAV9, respectively). With these coordinates, we generated AAV2 and AAV9 capsid oligomers comprising nine subunits or full capsids using VIPERdb Oligomer Generator^60^ and visualized them using PyMOL. We determined topological locations of each capsid amino acid in a tertiary and quaternary structure by visual inspection of the surface-rendered structural model of the AAV capsids using PyMOL.

### Statistical analysis

We assessed phenotypic differences between AAV*x*_*i*_ and AAV*y*_*k*_ by two-tailed Mann–Whitney *U*-test. We used a non-parametric test because Shapiro–Wilk test for normality revealed that *GNR* data sets with which PD (*x*_*i*_*, y*_*k*_) was determined (that is, *GNR_lt_VBC* (*x*_*ij*_*, y*_*k*_*, n*) and *GNR_rt_VBC* (*x*_*ij*_*, y*_*k*_*, n*) values) do not necessarily follow normal distribution. To assess correlation between two phenotypes, we used a *χ*^2^ test or Fisher’s exact test. We applied a Monte Carlo approach to determine statistical power of the Barcode-Seq analysis. Briefly, we obtained an actual Illumina sequence read number data set containing 100 different AAV9 clones AAV*x*_1*j*_ (*j*
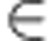
{1, 2,..., 100}) from the liver samples obtained from the three mice injected with the AAV-Serotype-VBCLib library. We then calculated *GNR_lt_VBC* (*x*_1*j*_*, y*_1_*, n*) and *GNR_rt_VBC* (*x*_1*j*_*, y*_1_*, n*) (*j*
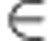
{1, 2,..., 100} and *n*∈{1, 2, 3}) and generated a GNR data set comprising 100 (AAV9 clones) × 2 (lt- and rt-VBCs) × 3 (mice)=600 *GNR_lt_VBC and GNR_rt_VBC* values. Using this GNR data set, we simulated the following 7 data sets showing an 0.125-, 0.25-, 0.5-, 1-, 2-, 4- and 8-fold increase by multiplying each *GNR_lt_VBC* (*x*_1*j*_*, y*_1_*, n*) and *GNR_rt_VBC* (*x*_1*j*_*, y*_1_*, n*) by a corresponding fold increase factor. We then randomly selected *no_of_ref* reference AAV clones from the onefold increase GNR data set (*no_of_ref* 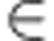
{3, 4, 5, …, 24}) and 2 AAV clones from each of the 0.125-, 0.25-, 0.5-, 1-, 2-, 4- and 8-fold GNR data sets from two or three mice, and statistically assessed a difference in these two selected subdatasets by two-tailed Mann–Whitney *U*-test. We performed this simulation 500 times and determined the statistical power of the analysis to detect each of 0.125-, 0.25-, 0.5-, 1-, 2-, 4- and 8-fold changes with *P<*0.05 (two-tailed Mann–Whitney *U*-test). We also performed the same power analysis using the randomly undersampled data sets generated from the Illumina sequencing read number data sets of 100 different AAV9 clones *x*_1*j*_ described above. Statistical algorithms were developed and implemented in Perl with CPAN modules at the Pittsburgh Supercomputing Center. We used all the values without exclusion for the simulation studies that validated the AAV Barcode-Seq analysis. For the assessment of the actual experimental data obtained by the AAV Barcode-Seq analysis, we excluded outliers showing values more than three times the interquartile range beyond the upper and lower quartiles.

### Clustering analyses

In the hierarchical clustering analysis, we used the Manhattan distance and the complete linkage method. We used R^61^ for computation and graphic output.

## Author contributions

H.N. conceived and designed the project. K.A., T.E., Y.K., M.V. and H.N. constructed plasmids. K.A., T.E. and Y.K. produced the AAV libraries. K.A. performed the *in vitro* and *in vivo* experiments with assistance from H.N. H.N. developed the algorithm for data analysis and wrote the computer scripts. K.A. and H.N. analysed the results and wrote the manuscript. All authors commented on the manuscript.

## Additional information

**Accession codes:** Sequences of plasmids pAAV9-SBBANN-VBCLib and pAAV2R585E-SBBXEB-VBCLib have been deposited in the GenBank nucleotide core database under the accession codes KF032296 and KF032297, respectively.

**How to cite this article:** Adachi, K. *et al.* Drawing a high-resolution functional map of adeno-associated virus capsid by massively parallel sequencing. *Nat. Commun.* 5:3075 doi: 10.1038/ncomms4075 (2014).

## Supplementary Material

Supplementary Information Supplementary Figures 1-10, Supplementary Tables 1-5, Supplementary Methods and Supplementary Reference

Supplementary Data 1 AAV Barcode-Seq data obtained with AAV-Serotype-VBCLib

Supplementary Data 2 AAV Barcode-Seq data obtained with AAV9-AA-VBCLib

Supplementary Data 3 AAV Barcode-Seq data obtained with AAV2R585E-HP-VBCLib

## Figures and Tables

**Table 1:** DNA-barcoded AAV virus libraries.

**Library name**	**AAV clones contained in the libraries**			**Amino acids to be investigated**	**Total no. of clones**
	**AAV strains**	**No. of mutants**	**No. of clones**		
AAV-Serotype-VBCLib					
	AAV9	Control	100		
	AAV2R585E	Control	10		
	AAV1	Wild type	2		
	AAV2	Wild type	2		
	AAV3	Wild type	2		
	AAV4*	Wild type	2		
	AAV5	Wild type	2		
	AAV6	Wild type	2		
	AAV7	Wild type	2		
	AAV8	Wild type	2		
	AAVrh10	Wild type	2		
	AAV1.9-1†	Hybrid	2		
	AAV1.9-3-5†	Hybrid	2		132
					
AAV9-AA-VBCLib-1					
	AAV9	Control	15		
	AAV2R585E	Control	15		
	AAV9 double alanine mutants	92	184 (2 each)	356–539	214
					
AAV9-AA-VBCLib-2					
	AAV9	Control	15		
	AAV2R585E	Control	15		
	AAV9 double alanine mutants	38	76 (2 each)	540–615	106
					
AAV9-AA-VBCLib-3					
	AAV9	Control	15		
	AAV2R585E	Control	15		
	AAV9 double alanine mutants	61	122 (2 each)	616–736	152
					
AAV9-AA-VBCLib-4					
	AAV9	Control	18		
	AAV2R585E	Control	18		
	AAV9 double alanine mutants	119	238 (2 each)		
	AAV2R585E hexapeptide mutants	11	22 (2 each)	See footnote‡	
	AAV2R585E.9-2	1	2		298
					
AAV2R585E-HP-VBCLib-1					
	AAV9	Control	15		
	AAV2R585E	Control	15		
	AAV2R585E hexapeptide mutants	66	132 (2 each)	441–484§	162
					
AAV2R585E-HP-VBCLib-2					
	AAV9	Control	15		
	AAV2R585E	Control	15		
	AAV2	Wild type	2		
	AAV2R585E hexapeptide mutants	59	177 (3 each)	571–604§	209

^*^We lost AAV4 viral particles significantly from the library during the viral stock purification procedure. Accordingly, Illumina sequence read numbers for AAV4 in the viral stock were several logs lower than others. Therefore, AAV4 data were not evaluated or included in the study.

^†^AAV1.9-1 and AAV1.9-3-5 are AAV1 and AAV9 hybrid capsids^44^.

^‡^The following 11 AAV2R585E hexapeptide mutants were included: 463-16000, 465-00700, 465-16000, 467-16000, 479-00009, 573-00780, 581-10000, 583-06000, 585-00080, 593-10000 and 595-10000.

^§^The amino-acid positions are provided based on AAV2 VP1.
