# Supplementary material for: Drawing a high-resolution functional map of adeno-associated virus capsid by massively parallel sequencing
Source: Nat Commun. 2014 Jan 17;5:3075. doi: 10.1038/ncomms4075 (PMC3941020; doi:10.1038/ncomms4075)
Supplement: Supplementary Information — Supplementary Figures 1-10, Supplementary Tables 1-5, Supplementary Methods and Supplementary Reference [file ncomms4075-s1.pdf]

SUPPLEMENTARY INFORMATION

Drawing a high-resolution functional map of adeno-associated virus capsid by massively parallel sequencing

Kei Adachi[1,3], Tatsuji Enoki[2], Yasuhiro Kawano[1,2], Michael Veraz[1] & Hiroyuki Nakai[1]

[1]Department of Molecular & Medical Genetics, Oregon Health and Science University School of Medicine, Portland, Oregon 97239, USA; [2]Takara Bio Inc. Otsu Shiga 520-2134, Japan.

CONTENTS

1. Supplementary Figures

- Supplementary Figure 1: Validation of the Barcode-Seq analysis
- Supplementary Figure 2: Statistical power of the AAV Barcode-Seq analysis
- Supplementary Figure 3: Mouse tissue transduction efficiencies of various AAV serotypes determined by AAV Barcode-Seq
- Supplementary Figure 4: Blood AAV concentration-time curves following intravenous injection of various AAV serotypes in adult C57BL/6 male mice
- Supplementary Figure 5: Sialic acid-mediated cell surface binding and transduction of various AAV serotypes determined by AAV Barcode-Seq
- Supplementary Figure 6: Cross-reactivity of anti-AAV9 neutralizing antibody to various AAV serotypes
- Supplementary Figure 7: A heatmap showing the result of a hierarchical clustering analysis of the *in vivo* transduction profiles of the liver-detargeted AAV9 mutants
- Supplementary Figure 8: Topological location of the AAV9 capsid amino-acid residues important for galactose binding
- Supplementary Figure 9: Blood AAV concentration-time curves following intravenous injection of AAV mutant libraries
- Supplementary Figure 10: Anti-AAV neutralizing antibody epitope mapping by AAV Barcode-Seq

2. Supplementary Tables

- Supplementary Table 1: Hexapeptide scanning AAV2R585E-derived mutants
- Supplementary Table 2: Vector genome copy numbers in various tissues of mice injected with AAV-CMV-lacZ vectors
- Supplementary Table 3: Transduction efficiency in the liver and heart of mice injected with various AAV-CMV-lacZ vectors
- Supplementary Table 4: Correlation between liver transduction and pharmacokinetic profiles of AAV9 mutants
- Supplementary Table 5: Correlation between liver transduction and pharmacokinetic profiles of AAV2R585E mutants

3. Supplementary Methods

- Cell culture experiments
- Plasmid construction
- Illumina sequencing
- Determination of the relative yields of virus production

4. Supplementary Reference

1. SUPPLEMENTARY FIGURES

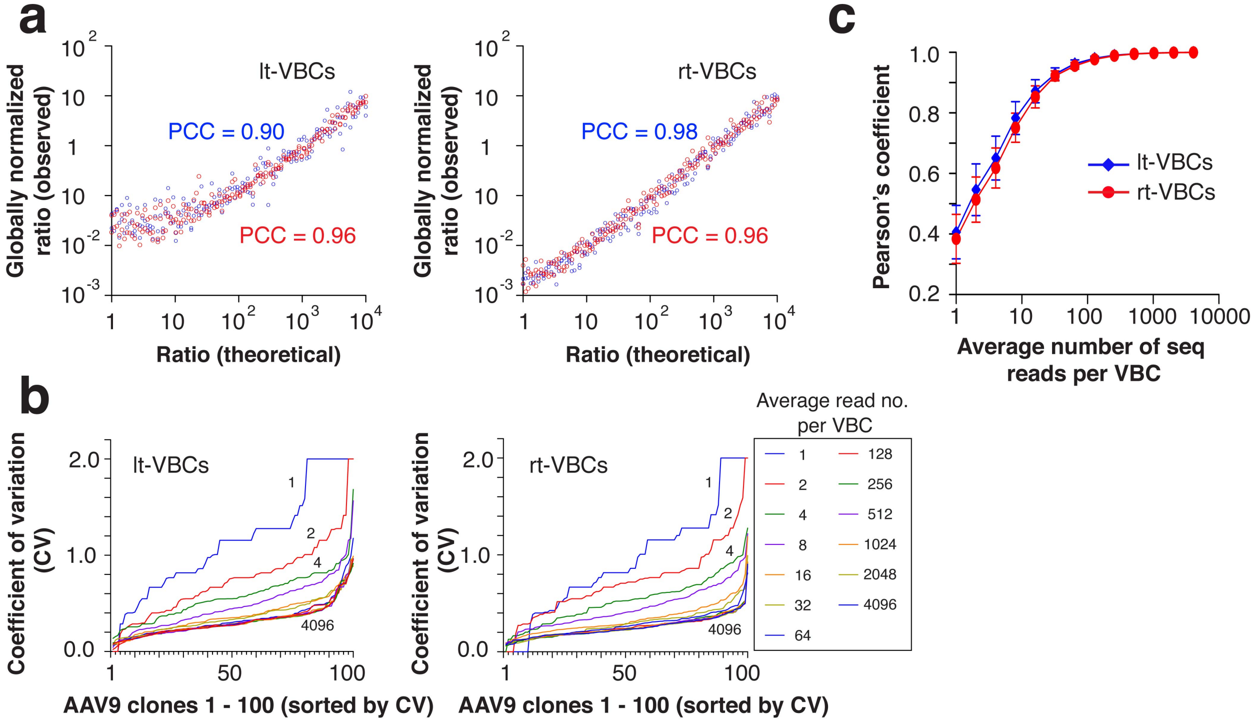

Supplementary Fig. 1

Validation of the Barcode-Seq analysis. **(a)** Correlation between the relative abundance of DNA templates and Illumina sequencing read numbers. We generated two types of pr-VBC libraries, Lib-1 and Lib-2. Each library was a mixture of the same set of 100 different pr-VBCs. We mixed these pr-VBCs at an equimolar ratio in Lib-1, while we mixed the pr-VBCs at 100 different known concentrations in Lib-2. The lowest and highest concentrations of the 100 pr-VBCs in Lib-2 were 1 and 10,000 relative concentration units; and the concentrations were serially increased from the lowest to the highest by a factor of $10^{4/99} = 1.0975$. Using these libraries as templates, we PCR-amplified lt- and rt-VBCs separately, and performed Illumina sequencing of the VBC-PCR amplicons in duplicate. We determined globally normalized Lib-2-to-Lib-1 Illumina sequence read number ratios (Y-axis) and plotted them against theoretical Lib-2-to-Lib-1 ratios calculated by the known concentrations of pr-VBCs (X-axis). Blue and red dots indicate two different sets of duplicated experiments. PCC, Pearson's correlation coefficient. **(b)** Coefficient of variation (CV) of globally normalized Illumina sequence read numbers for each of the 100 lt- and rt-VBCs in quadruplicated analyses of undersampled data sets showing various Illumina sequencing depths (*i.e.*, average number of sequence reads per VBC). The lt- and rt-VBCs were sorted by their CV values from the smallest to the largest. In this analysis, we PCR-amplified lt- and rt-VBCs in quadruplicate using the same DNA template containing a mixture of 100 different pr-VBCs, Illumina-sequenced the VBC-PCR amplicons, and obtained Illumina sequence read numbers for each VBC-PCR amplicon. We then performed an undersampling

simulation study[38]. CV in the quadruplicated data sets was 0.31 on average when each VBC was contained 4,096 times on average in a data set. The data reproducibility did not deteriorate in smaller data sets containing ≥64 reads per VBC on average. **(c)** Data correlation between an undersampled data set (*i.e.*, ≤4096 reads per VBC on average) and a full-size data set (*i.e.*, 4,571 - 11,256 reads per VBC on average). PCC was determined by repeating the undersampling simulation 100 times for each sample size. Error bars are s.d.

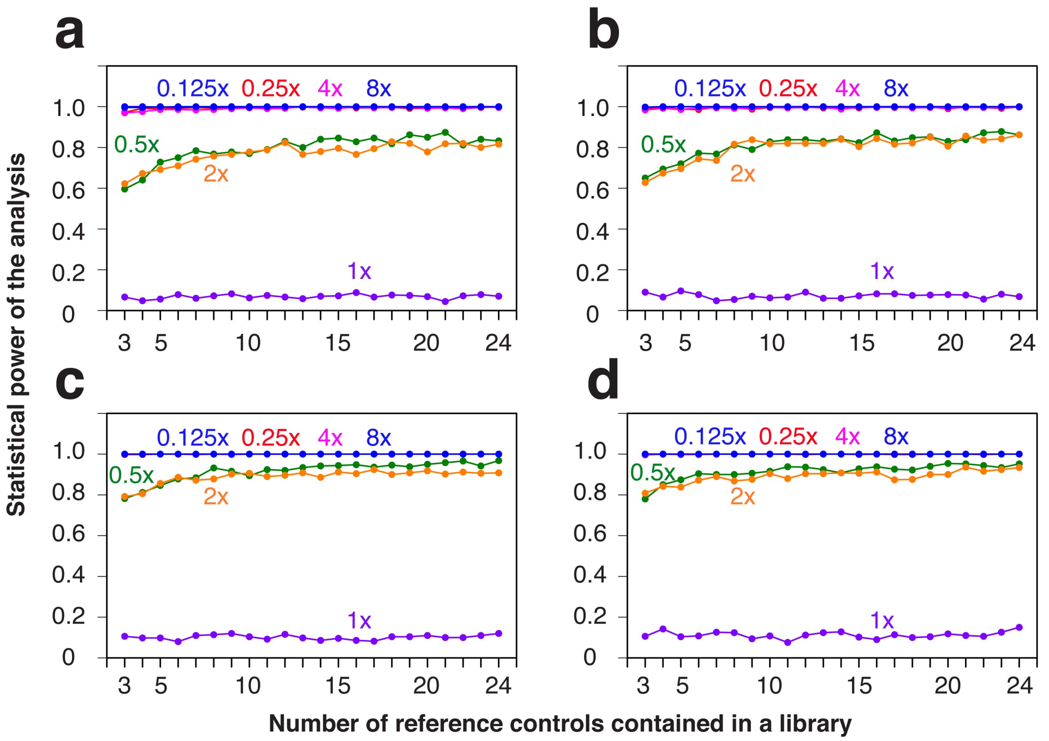

Supplementary Fig. 2

Statistical power of the AAV Barcode-Seq analysis. We determined the power of the statistical analysis in analyzing hepatic transduction as a function of the number of reference controls. To determine the power, we performed a Monte Carlo simulation study using an actual experimental data set of murine liver transduction efficiencies of 100 AAV9 clones, which were exactly the same wild-type AAV9 viruses except for the DNA barcodes embedded in their viral genome. The simulation was run 500 times. To obtain this experimental data set, we injected three adult C57BL/6 male mice with 1×10^{12} vector genomes (vg) of AAV-Serotype-VBCLib via the tail vein, harvested liver tissues 6 weeks post-injection, recovered total liver DNA, and performed the AAV Barcode-Seq analysis. CVs of liver transduction efficiencies of the 100 AAV9 clones in these three mice, determined by Barcode-Seq, were 1.03, 0.99 and 0.59 for lt-VBCs and 0.77, 0.53 and 0.54 for rt-VBCs in mice 1, 2 and 3, respectively. (**a, b**) A power analysis was performed on an undersampled data set in which the average sequence read numbers of reference controls were 64 and 4096 for Panels a and b, respectively. This simulated experiment used a library containing 2 clones per mutant and was done in duplicate. (**c, d**) The same analysis was performed on an undersampled data set with the average sequence read numbers of reference controls being 64 and 4096 for Panels c and d, respectively. This simulated experiment used a library containing 2 clones per mutant and was done in triplicate. We used two-tailed Mann-Whitney *U*-test.

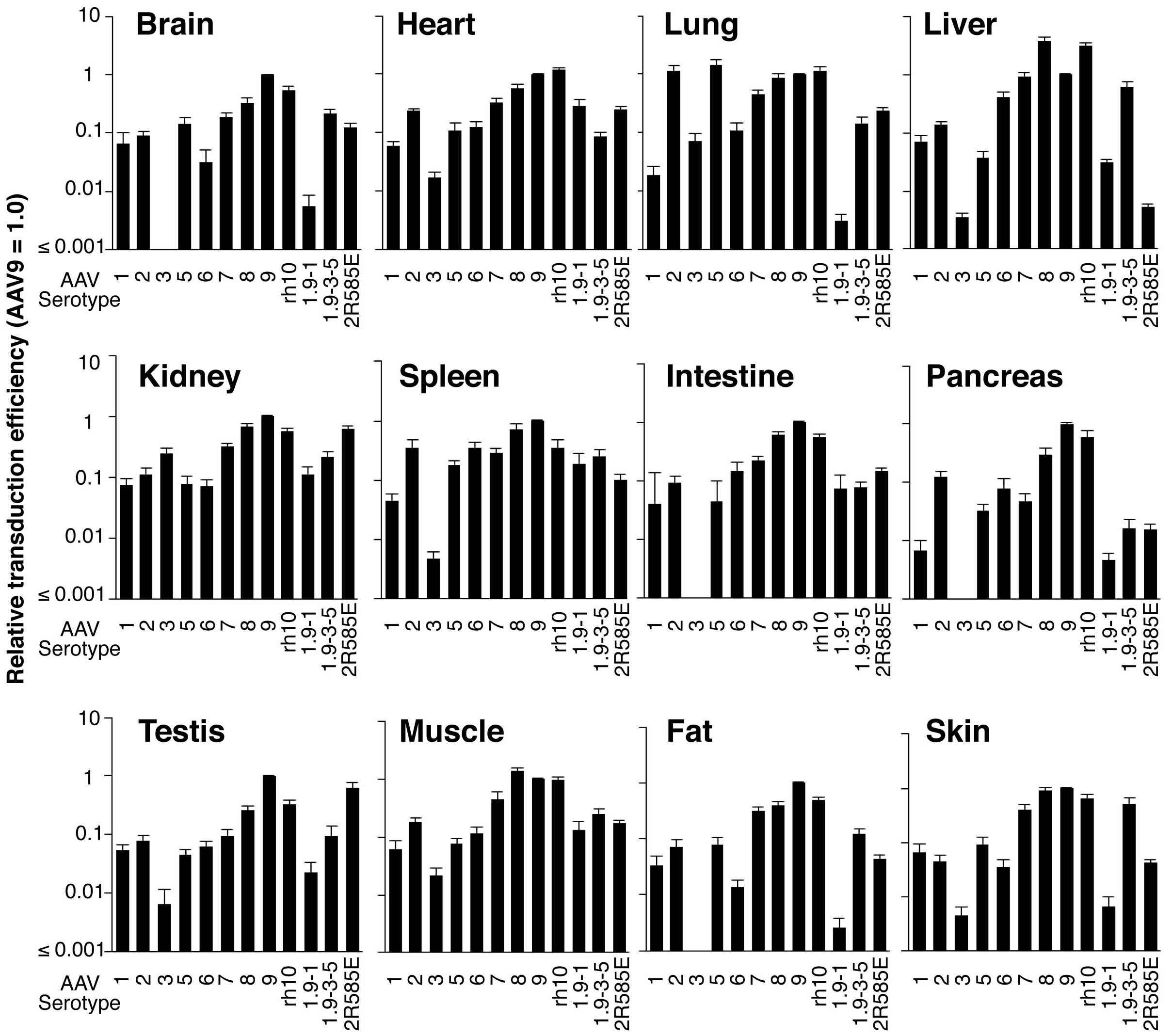

Supplementary Fig. 3

Mouse tissue transduction efficiencies of various AAV serotypes determined by AAV Barcode-Seq. We injected adult C57BL/6 male mice with AAV-Serotype-VBCLib at a dose of 1 $\times 10^{12}$ vg per mouse (n=3) via the tail vein. We harvested 12 major tissues 6 weeks post-injection, extracted total DNA from each tissue, and performed the AAV Barcode-Seq analysis. All the transduction efficiencies are normalized with those of AAV9 in each tissue. Error bars represent s.e.m. Please note that AAV2 transduction efficiency in the lung was comparable to that of AAV9. This was unexpected but was reproducible with the AAV2R585E-HP-VBCLib-2 library, which also contained AAV2 clones.

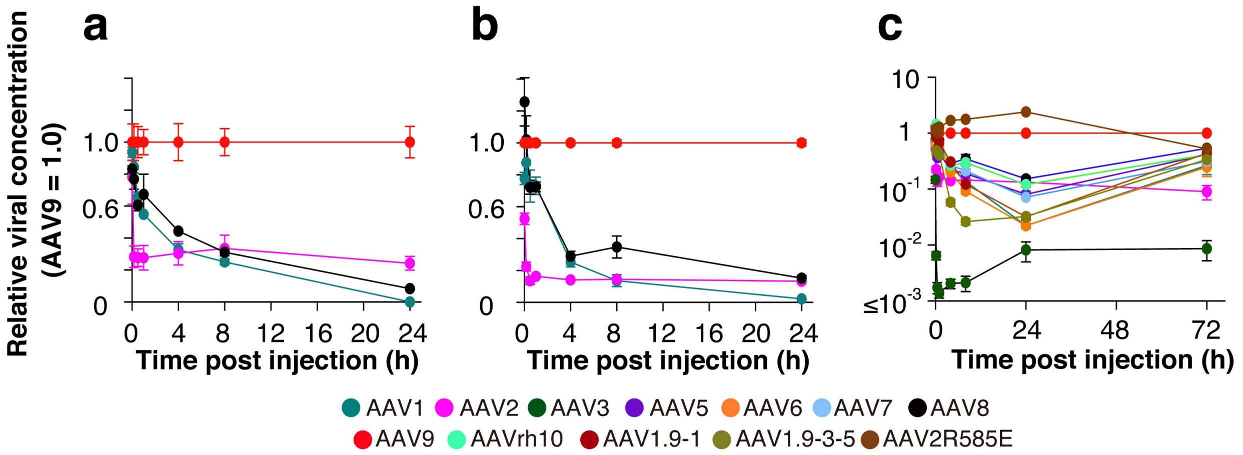

Supplementary Fig. 4

Blood AAV concentration-time curves following intravenous injection of various AAV serotypes into adult C57BL/6 male mice. (**a**) Pharmacokinetic profiles of AAV1, 2, 8 and 9 determined by a conventional approach in our previous study[44]. (**b**) Pharmacokinetic profiles of AAV1, 2, 8 and 9 determined by AAV Barcode-Seq in this study. We injected adult C57BL/6 male mice with AAV-Serotype-VBCLib at a dose of 1×10^{13} vg per kg (n=3) via the tail vein as a bolus, and determined relative AAV concentrations in the blood by the AAV Barcode-Seq analysis. (**c**) Pharmacokinetic profiles of all the AAV strains contained in AAV-Serotype-VBCLib. All values are presented as those normalized by the values of AAV9. Error bars represent s.e.m.

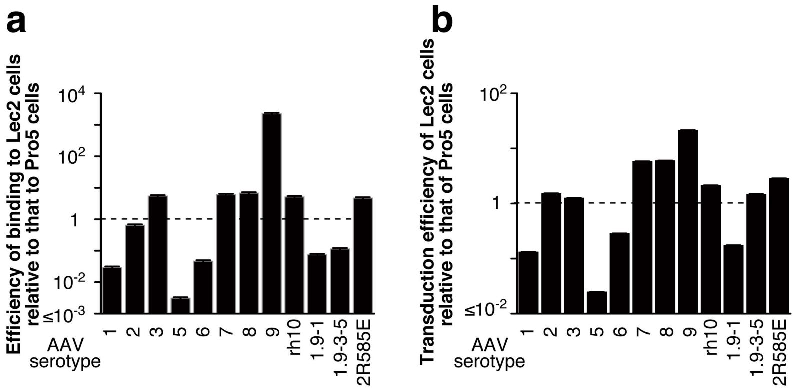

Supplementary Fig. 5

Sialic acid-mediated cell surface binding and transduction of various AAV serotypes determined by AAV Barcode-Seq. We applied AAV-Serotype-VBCLib to CHO Pro5 and Lec2 cells at an MOI of 10^5 and determined cell surface binding and *in vitro* transduction efficiencies of each AAV strain by AAV Barcode-Seq (n=3 per group). In the same set of experiments, we also applied double-stranded (ds) AAV2-CMV-GFP vector to Pro5 and Lec2 cells, and determined quantities of cell surface-bound AAV2 particles by qPCR and transduction efficiencies by flow cytometry. Due to the relative nature of the AAV Barcode-Seq analysis within a sample, the latter set of data was necessary to compare the results between Pro5 and Lec2 cells and determine Lec2 cell binding or transduction efficiency relative to that of Pro5 cells. (**a**) Results of the cell surface binding assay. (**b**) Results of the cell transduction assay. Error bars, which are barely seen, represent s.e.m.

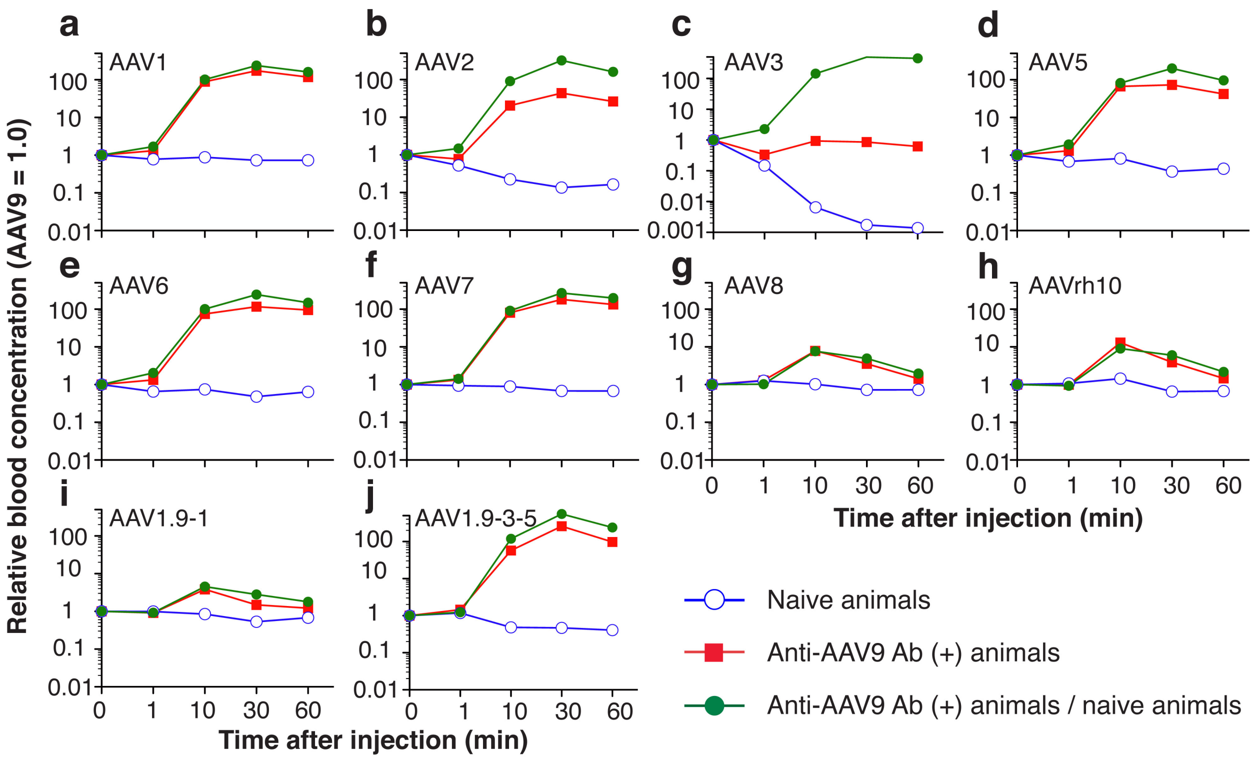

Supplementary Fig. 6

Cross-reactivity of anti-AAV9 neutralizing antibody to various AAV serotypes. We intravenously injected 1 × 10^{13} vg per kg of AAV-Serotype-VBCLib as a bolus into naïve adult C57BL/6 male mice and mice pre-immunized with AAV9-CMV-lacZ vector (n=3 per group), and determined relative blood concentrations of each serotype by AAV Barcode-Seq over 60 minutes. Blue and red lines show blood vector concentrations relative to that of AAV9 in naive mice and the anti-AAV9 antibody-harboring mice, respectively. Green lines indicate red-line-value-to-blue-line-value ratios. In anti-AAV9 antibody-harboring mice, because AAV9 particles are quickly cleared from the blood circulation, the relative blood concentrations of AAV serotypes that are not neutralized by anti-AAV9 antibody would exhibit a dramatic increase compared to those of AAV9, showing strikingly elevated green line values. In contrast, AAV serotypes that are neutralized would exhibit a pharmacokinetic profile similar to that of AAV9. Panels a, b, c, d, e, f, and j exhibit a pattern indicating no cross-reactivity while Panels g, h and i show a pattern indicating positive cross-reactivity. Please note that AAV3 is cleared from the blood circulation much faster than AAV9 and other serotypes (**Supplementary Fig. 4c**), making its pharmacokinetic profiles (Panel c) distinct from the others. As for AAV8 and AAVrh10, we observed mouse-to-mouse differences in the anti-AAV9 neutralizing antibody reactivity. Therefore, we collected the data from a total of 5 mice, among which the same 3 mice showed positive cross-reactivity to both AAV8 and AAVrh10 while the same 2 mice exhibited a negative pattern to the both. For these two serotypes, only the data showing positive reactivity are shown in the graphs.

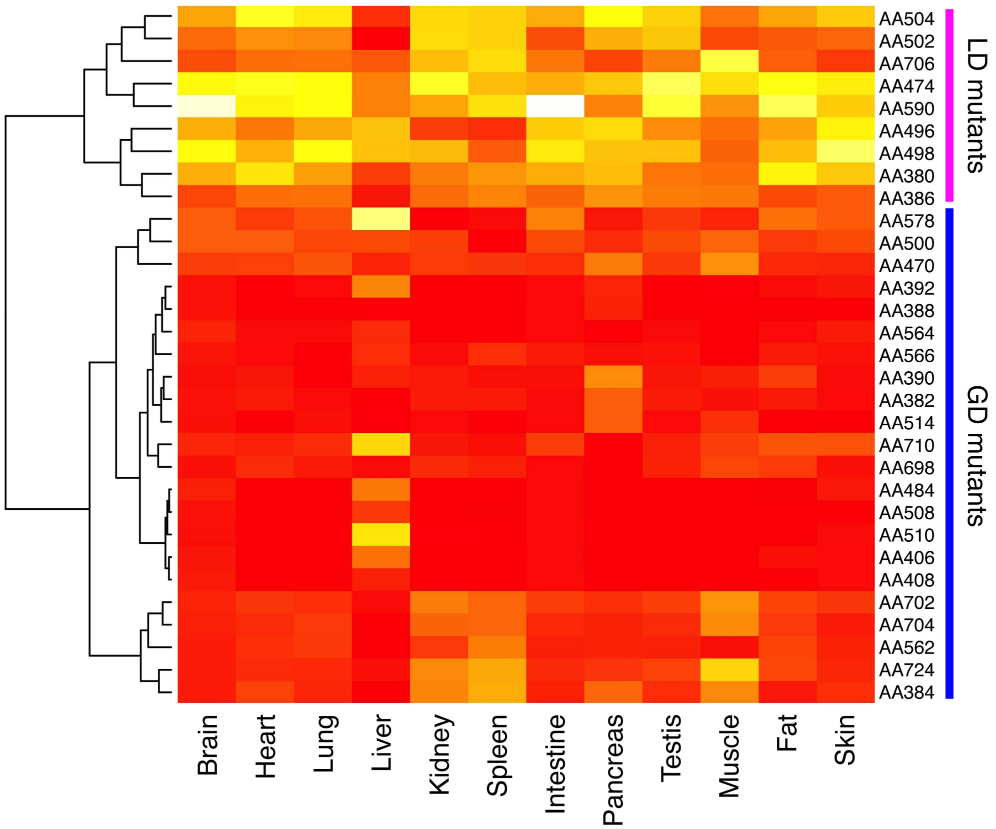

Supplementary Fig. 7

A heatmap showing the result of a hierarchical clustering analysis (complete linkage method, Manhattan distance) of the *in vivo* transduction profiles of the 31 liver-detargeted AAV9 mutants. The data were scaled by column. They are grouped into two phenotypically distinct groups: LD mutants and GD mutants. The LD mutants mainly detarget the liver while GD mutants show impaired transduction in many tissues in addition to the liver.

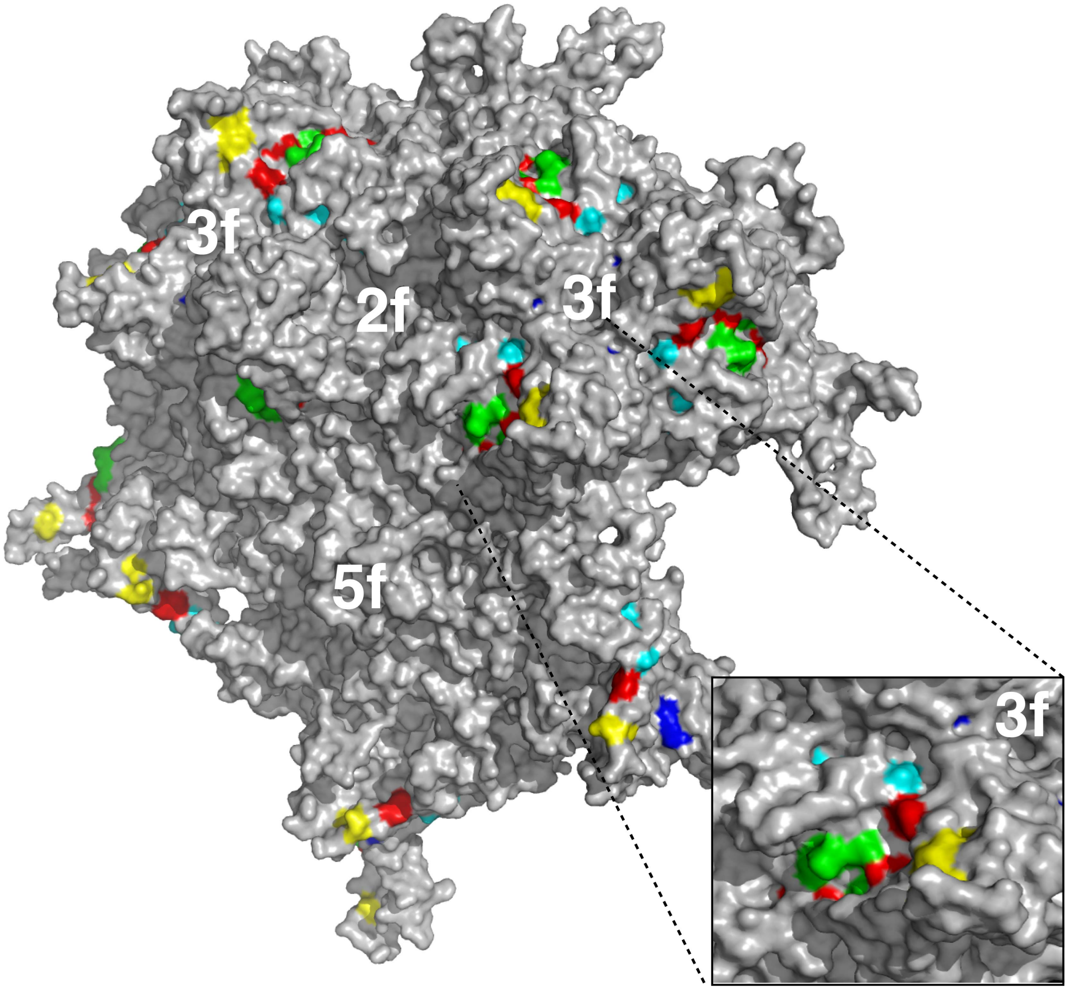

Supplementary Fig. 8

Topological location of the AAV9 capsid amino-acid residues important for galactose binding. Green (V465, P468, S469 and N470), yellow (I451, E500 and F501), cyan (L382, N383, R514 and N515) amino acids form three small clusters. These clusters are bridged by a cluster of red conserved amino acids (I440, D441, Y446, L447, W503), forming a larger cluster inside a pocket between the three- and five-fold symmetry axes. R485 (blue) resides on the inner wall of the threefold protrusions. The other 9 amino acids important for galactose binding are not exposed on the surface.

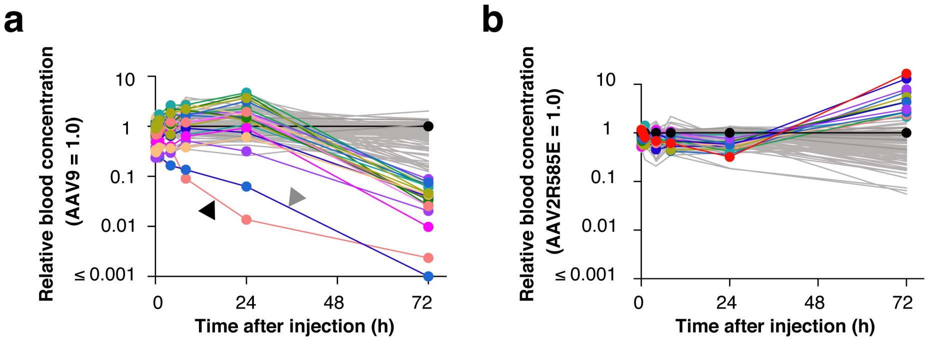

Supplementary Fig. 9

Blood AAV concentration-time curves following intravenous injection of AAV mutant libraries; AAV9-AA-VBCLib (Panel a) and AAV2R585E-HP-VBCLib (Panel b). We injected 1 × 10^{13} vg per kg of each AAV library into adult C57BL/6 male mice as a bolus (*n*=2 per library), and determined blood AAV concentrations of each mutant by AAV Barcode-Seq over 72 hours post-injection. All the values are normalized with those of reference controls. (**a**) All the 119 AAV9 mutants. The 20 mutants showing an LP phenotype are indicated with colors. The other mutants are shown with gray lines. AAV9Y484A/R485A and AAV9S508A/W509A are indicated with black and gray arrowheads, respectively. These two mutants are among those that showed a substantial increase in binding to CHO Pro5 cells (**Fig. 3d**). The wild-type AAV9 is indicated with a black line. (**b**) All the 117 AAV2R585E mutants. The 13 AAV2R585E mutants exhibiting a DC phenotype are indicated with colors besides red. The other mutants are shown with gray lines. AAV2R585E and the wild-type AAV9 are indicated with black and red lines, respectively. Graph legends for lines and error bars are omitted.

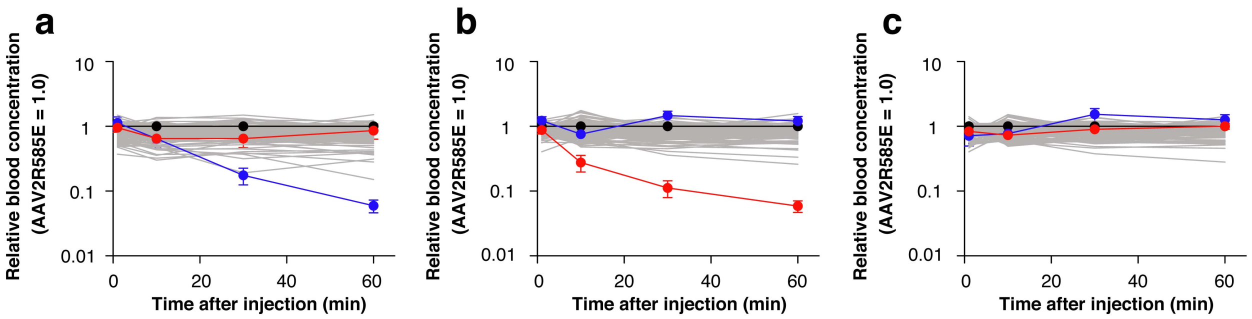

Supplementary Fig. 10

Anti-AAV neutralizing antibody epitope mapping by AAV Barcode-Seq. Pharmacokinetic profiles of the 117 hexapeptide scanning AAV2R585E mutants following intravenous injection of AAV2R585E-HP-VBCLib are shown. We injected adult C57BL/6 male mice harboring anti-AAV1 (Panel a) or anti-AAV9 (Panel b) neutralizing antibody with the library intravenously as a bolus at a dose of 1×10^{13} vg per kg (*n*=3 per library). AAV2R585E-HP-VBCLib-injected naïve animals (*n*=2 per library) used for **Supplementary Fig. 9b** served as the control (Panel c). We determined blood AAV concentrations of each AAV mutant relative to those of AAV2R585E by AAV Barcode-Seq. Only the results of AAV2R585E, 451-16000, and 451-00009 are highlighted with black, blue and red lines, respectively. The results of all the other 115 mutants are shown with gray lines. The blue and red mutants exhibited significantly accelerated blood clearance only in anti-AAV1 and anti-AAV9 neutralizing antibody-harboring animals, respectively. This was not observed in the naïve animals (Panel c). In the 451-16000 and 451-00009 mutants, the native AAV2R585E sequence 451-PSGTTT-456 was replaced with -QSGSAQ- derived from the AAV1 capsid and -GSGQN- derived from the AAV9 capsid, respectively. This indicates that QSGSAQ and GSGQN are anti-AAV1 and anti-AAV9 capsid antibody epitopes, respectively. Graph legends for lines and s.e.m. error bars are omitted except for the highlighted three AAV strains.

2. SUPPLEMENTARY TABLES

Supplementary Table 1 Hexapeptide scanning AAV2R585E-derived mutants

Name of mutant*	Amino-acid substitutions in addition to R585E
441-00700	S446A
441-16000	S446N
443-00009	R447K
445-00009	R447K/N449I/T450N
445-00080	N449Q
445-00700	S446A/N449Q/T450S
445-16000	S446N/N449Q/T450N
447-00009	R447K/N449I/T450N/P451G/S452
447-00080	N449Q/P451T/S452G
447-00700	N449Q/T450S/P451N/S452G
447-16000	N449Q/T450N/P451Q
449-00009	N449I/T450N/P451G/S452/G453S/T454G
449-00700	N449Q/T450S/P451N/S452G
449-16000	N449Q/T450N/P451Q/T454S
451-00009	P451G/S452/G453S/T454G/T455Q/T456N
451-00080	P451T/S452G/T455A/T456N
451-00700	P451N/S452G/T455A/T456G
451-16000	P451Q/T454S/T455A/T456Q
453-00009	G453S/T454G/T455Q/T456N/S458Q
453-00080	T455A/T456N/Q457T/S458Q
453-00700	T455A/T456G/Q457N/S458R
453-16000	T454S/T455A/T456Q/Q457N/S458K
455-00009	T455Q/T456N/S458Q/R459T
455-00080	T455A/T456N/Q457T/S458Q/R459T
455-00700	T455A/T456G/Q457N/S458R/R459E
455-16000	T455A/T456Q/Q457N/S458K/R459D
457-00009	S458Q/R459T/Q461K
457-00080	Q457T/S458Q/R459T/Q461G
457-00700	Q457N/S458R/R459E
457-16000	Q457N/S458K/R459D/Q461L
459-00009	R459T/Q461K/Q464V
459-00080	R459T/Q461G
459-00700	R459E/S463Y
459-16000	R459D/Q461L/Q464R
461-00009	Q461K/Q464V
461-00080	Q461G/A465G
461-00700	S463Y/A465G
461-16000	Q461L/Q464R/A465G/G466S
463-00009	Q464V/A467P
463-00080	A465G/A467P/S468N
463-00700	S463Y/A465G/A467P
463-16000	Q464R/A465G/G466S/A467P/S468A
465-00009 (R585E.9-4†)	A467P/D469N/I470M

*The following system is used to name the hexapeptide scanning AAV2R585E mutants. The left three digits indicate the first amino-acid position of the hexapeptide based on AAV2 VP1. The right five digits indicate AAV serotype from which each hexapeptide is derived: 10000, AAV1; 06000, AAV6; 00700, AAV7; 00080, AAV8; and 00009, AAV9. When a hexapeptide amino-acid sequence is shared with multiple serotypes, the right five digits have more than one positive integer.

† Alternative names used in this study.

465-00080	A465G/A467P/S468N/D469T/I470M
465-00700	A465G/A467P/D469T/I470M
465-16000	A465G/G466S/A467P/S468A/D469G/I470M
467-00009 (R585E.9-5[†])	A467P/D469N/I470M/R471A/D472V
467-00080	A467P/S468N/D469T/I470M/R471A/D472N
467-00700	A467P/D469T/I470M/R471A/D472E
467-16000	A467P/S468A/D469G/I470M/R471S/D472V
469-00009	D469N/I470M/R471A/D472V/S474G
469-00080	D469T/I470M/R471A/D472N/S474A
469-00700	D469T/I470M/R471A/D472E/S474A
469-16000	D469G/I470M/R471S/D472V/S474P
471-00009	R471A/D472V/S474G
471-00080	R471A/D472N/S474A/R475K
471-00700	R471A/D472E/S474A/R475K
471-16000	R471S/D472V/S474P/R475K
473-00009	S474G/W477Y/L478I
473-00780	S474A/R475K
473-16000	S474P/R475K
475-00009	W477Y/L478I
475-16780	R475K
477-00009	W477Y/L478I/C482S
479-00009	C482S
479-00700	Y483F
571-00009	Q575S
571-00780	Q575E
571-16000	Q575R/Y576F
573-00009	Q575S/S578Q
573-00780	Q575E/S578I
573-16000	Q575R/Y576F/S578T
575-00009	Q575S/S578Q/S580A
575-00080	Q575E/S578I/S580A
575-16000	Q575R/Y576F/S578T/S580A
577-00009	S578Q/S580A
577-00080	S578I/S580A/T581D
577-00700	S578I/T581S
577-16000	S578T/S580A/T581V
579-00009	S580A/L583H
579-00080	S580A/T581D
579-00700	T581S
579-06000	S580A/T581V
579-10000	S580A/T581V/L583F
581-00009	L583H/E585S/G586A
581-00080	T581D/E585Q/G586Q
581-00700	T581S/E585A/G586A
581-06000	T581V/E585S/G586S
581-10000	T581V/L583F/E585S/G586S
583-00009	L583H/E585S/G586A/N587Q/R588A
583-00080	E585Q/G586Q/R588T
583-00700	E585A/G586A/R588T
583-06000	E585S/G586S/N587S/R588T
583-10000	L583F/E585S/G586S/N587S/R588T
585-00009 (2i9[‡])	E585S/G586A/N587Q/R588A
585-00080 (2i8[‡])	E585Q/G586Q/R588T/Q589A/A590P

‡ Alternative names used by Asokan et al[33].

585-00700 (2i7‡)	E585A/G586A/R588T/Q589A
585-16000 (2i1‡)	E585S/G586S/N587S/R588T/Q589D/A590P
587-00009	N587Q/R588A/A591Q
587-00080	R588T/Q589A/A590P/A591Q/T592I
587-00700	R588T/Q589A/A591Q
587-16000	N587S/R588T/Q589D/A590P
589-00009	A591Q/A593G/D594W
589-00080	Q589A/A590P/A591Q/T592I/A593G/D594T
589-00700	Q589A/A591Q/A593Q/D594V
589-16000	Q589D/A590P/A593G/
591-00009	A591Q/A593G/D594W/N596Q
591-00080	A591Q/T592I/A593G/D594T
591-00700	A591Q/A593Q/D594V
591-16000	A593G/N596H
593-00009	A593G/D594W/N596Q/T597N
593-00080	A593G/D594T/T597S
593-00700	A593Q/D594V/T597N
593-06000	A593G/N596H/T597V/Q598M
593-10000	A593G/N596H/T597A/Q598M
595-00009	N596Q/T597N/V600I
595-00080	T597S/V600A
595-00700	T597N/V600A
595-06000	N596H/T597V/Q598M/V600A
595-10000	N596H/T597A/Q598M/V600A
597-00009	T597N/V600I
597-06000	T597V/Q598M/V600A
597-10000	T597A/Q598M/V600A
599-00009	V600I
599-16780	V600A

Supplementary Table 2 Vector genome copy numbers in various tissues of mice injected with AAV-CMV-lacZ vectors

Vector	Vector genome copy number[*,†]								
	C57BL/6 (11 days p.i.)[‡]		C57BL/6 Rag1-/- (6 weeks p.i.)[‡]						
	Liver 1×10^{12}	Liver 3×10^{11}	Liver 1×10^{12}	Liver 3×10^{11}	Heart 1×10^{12}	Heart 3×10^{11}	Pancreas 1×10^{12}	Kidney 1×10^{12}	Brain 1×10^{12}
AAV9	192.3 ± 21.2	56.1 ± 4.1[§]	128.6 ± 36.5	44.6 ± 8.4	9.3 ± 3.2	n.d.	3.8 ± 1.3	1.7 ± 0.5	0.5 ± 0.0
P504A/G505A	0.2 ± 0.0	0.1 ± 0.0	0.6 ± 0.0	0.2 ± 0.0	8.6 ± 1.3	n.d.	0.1 ± 0.0	5.4 ± 1.5	0.3 ± 0.1
N562A/E563A	0.3 ± 0.0	0.1 ± 0.0	0.6 ± 0.1	0.1 ± 0.0	0.3 ± 0.1	n.d.	0.1 ± 0.0	< 0.05	< 0.01
Q590A	1.2 ± 0.2	0.7 ± 0.4	5.5 ± 1.4	0.9 ± 0.3	4.8 ± 1.1	n.d.	0.5 ± 0.1	6.1 ± 0.5	0.2 ± 0.1
AAV2R585E	0.3 ± 0.1	0.1 ± 0.0	0.3 ± 0.0	0.1 ± 0.0	7.8 ± 2.7	n.d.	1.0 ± 0.2	n.d.	n.d.
463-16000	2.3 ± 0.3	0.7 ± 0.4	7.1 ± 1.2	1.3 ± 0.5	10.1 ± 1.4	n.d.	0.2 ± 0.0	n.d.	n.d.
AAV2R585E.9-2	34.8 ± 8.0	9.8 ± 0.8	27.7 ± 5.7	9.4 ± 1.2	13.0 ± 0.3	4.1 ± 0.8	1.8 ± 0.6	n.d.	n.d.
AAV2R585E.9-2 mtTG	n.d.	n.d.	5.8 ± 2.1	2.3 ± 0.5	9.5 ± 2.0	3.2 ± 0.9	n.d.	n.d.	n.d.
AAV2R585E.9-2mtQ	n.d.	n.d.	18.9 ± 4.9	6.5 ± 1.7	11.1 ± 2.1	6.6 ± 1.7	n.d.	n.d.	n.d.
AAV2R585E.9-2mtTGQ	n.d.	n.d.	0.7 ± 0.2	0.3 ± 0.1	5.0 ± 1.7	3.2 ± 0.9	n.d.	n.d.	n.d.

[*]Vector genome copy numbers were determined by Southern blot analysis (livers except for 9-2mtTG, 9-2mtQ and 9-2mtTGQ) or qPCR (non-hepatic tissues and some of the liver samples as indicated).

[†]Values indicate double-stranded vector genome copy numbers per diploid genomic equivalent (ds-vg per dge).

[‡] We injected either C57BL/6 or C57BL/6 Rag1-/- male mice with AAV-CMV-lacZ vector (the wild-type AAV9, AAV9-derived mutants or AAV2R585E-derived mutants) at a dose of 3×10^{11} or 1×10^{12} vg per mouse. We determined tissue transduction efficiencies at 11 days or 6 weeks post-injection (p.i.) as indicated.

[§] We also determined the vector genome copy numbers by qPCR. The result was 102.8 ± 27.5 ds-vg per dge.

n.d., not done.

All the values are mean ± s.e.m. (*n*=3 per group).

Supplementary Table 3   Transduction efficiency in the liver and heart of mice injected with various AAV-CMV-lacZ vectors

Vector	X-Gal staining (%)							
	C57BL/6 (11 days p.i.)*				C57BL/6 Rag1-/- (6 weeks p.i.)*			
	Liver† 1×10^{12}	Liver 3×10^{11}	Heart 1×10^{12}	Heart 3×10^{11}	Liver 1×10^{12}	Liver 3×10^{11}	Heart 1×10^{12}	Heart 3×10^{11}
AAV9	39.5 ± 7.0	14.1 ± 1.9	89.5 ± 1.0	67.3 ± 6.1	20.0 ± 7.0	16.5 ± 5.6	98.1 ± 0.1	83.6 ± 3.5
P504A/G505A	0.8 ± 0.2	0.4 ± 0.1	33.7 ± 3.2	9.4 ± 1.9	1.3 ± 0.0	0.4 ± 0.1	77.1 ± 5.6	38.5 ± 4.2
N562A/E563A	0.9 ± 0.2	0.2 ± 0.1	0.2 ± 0.1	0.0 ± 0.0	0.9 ± 0.0	0.3 ± 0.0	0.4 ± 0.2	0.1 ± 0.0
Q590A	3.1 ± 0.6	0.9 ± 0.5	66.8 ± 7.5	15.1 ± 1.1	5.2 ± 0.7	2.5 ± 0.6	95.1 ± 1.8	58.9 ± 4.3
AAV2R585E	1.0 ± 0.6	0.1 ± 0.1	57.7 ± 0.9	9.3 ± 1.8	1.0 ± 0.2	0.3 ± 0.0	90.1 ± 1.6	62.8 ± 1.2
463-16000	3.1 ± 0.6	1.9 ± 1.4	50.5 ± 2.0	19.4 ± 3.6	6.9 ± 0.5	3.8 ± 0.4	94.7 ± 1.7	56.1 ± 4.7
AAV2R585E.9-2	18.6 ± 4.5	11.6 ± 2.6	90.9 ± 1.4	53.7 ± 5.4	13.9 ± 2.2	6.0 ± 1.7	96.2 ± 1.0	72.5 ± 11.8
AAV2R585E.9-2mtTG	1.0 ± 0.5	0.4 ± 0.1	52.2 ± 3.0	16.8 ± 2.0	1.4 ± 0.3	0.5 ± 0.1	91.4 ± 1.7	63.1 ± 1.8
AAV2R585E.9-2mtQ	2.0 ± 0.3	0.9 ± 0.2	60.2 ± 4.8	50.3 ± 6.7	6.5 ± 1.0	2.8 ± 1.0	94.0 ± 2.5	69.0 ± 2.7
AAV2R585E.9-2mtTGQ	0.1 ± 0.1	0.0 ± 0.0	18.8 ± 3.1	3.3 ± 0.8	0.2 ± 0.1	0.0 ± 0.0.	83.3 ± 3.9	33.3 ± 4.0

*We injected either C57BL/6 or C57BL/6 Rag1-/- male mice with AAV-CMV-lacZ vector at a dose of 3×10^{11} or 1×10^{12} vg per mouse. We determined transduction efficiencies at 11 days (C57BL/6) or 6 weeks (C57BL/6 Rag1-/-) post-injection (p.i.) by X-Gal staining as previously described[40].

†Please note that transduction efficiencies determined by transgene expression (this table) and those determined by vector genome copy numbers (Supplementary Table 2) become disproportionate in the livers transduced at low levels as we have previously reported, presumably due to an increase in vector genome specific activity at low vector genome copy numbers per cell[40, 44].

All the values are mean ± s.e.m. (*n*=3 per group).

Supplementary Table 4 Correlation between liver transduction and pharmacokinetic profiles of AAV9 mutants

Pharmacokinetic phenotype*	Liver-detargeting phenotype†		Total
	LD or GD	non-LD or GD	
LP	18	2	20
Non-LP	13	86	99
Total	31	88	119

*LP is a pharmacokinetic phenotype showing a >90% decrease in the blood concentration of AAV particles 72 hours post-injection, compared to the wild-type AAV9.

† LD and GD are liver-detargeting phenotype showing attenuated transduction mainly in the liver (LD) and also in many of non-hepatic tissues (GD).

Supplementary Table 5 Correlation between liver transduction and pharmacokinetic profiles of AAV2R585E mutants

Pharmacokinetic phenotype*	Liver-transduction phenotype†		Total
	Enhanced	Not enhanced	
DC	12	1	13
Non-DC	9	95	104
Total	21	96	117

*DC (Delayed Clearance) mutants are those showing a >2-fold increase in the blood concentration of AAV particles 72 hours post-injection, compared to the AAV2R585E.

†"Enhanced" mutants are those showing a >2-fold increase in liver transduction compared to the AAV2R585E.

3. SUPPLEMENTARY METHODS

Cell culture experiments

We seeded cells on 24 or 96-well plates one day before each experiment, and infected cells with AAV libraries or dsAAV-CMV-GFP at the indicated MOIs. For the AAV Barcode-Seq experiments, we harvested cells 48 h after infection for the downstream analysis. In the experiments using dsAAV-CMV-GFP, we assessed transduction efficiencies 48 h after infection by observing cells under an EVOS inverted fluorescence microscope and by flow cytometry using a FACSCalibur (Becton Dickinson, Franklin Lakes, NJ). In the flow cytometry analysis, we counted at least 5000 cells to determine transduction efficiencies. For the cell surface binding assay, we seeded cells on 24-well plates. Next day, we pre-incubated cells with a complete medium at 4 °C for 1 h. We then applied AAV vectors to cells at an MOI of 10^4 and co-incubated cells and AAV vectors at 4 °C for 1 h. We then washed cells with a pre-chilled complete medium 3 times to remove unbound AAV particles, harvested cells in DNA extraction buffer (50mM Tris-HCl (pH 8.0), 20mM EDTA, 0.1M NaCl, 1% SDS), and extracted total DNA including AAV viral genome DNA in cell surface-bound viral particles. For qPCR assay, we mixed 10 ng of total DNA with Power SYBR Green Master Mix Reagents (Life Technologies) and PCR primers (10 pmol each per reaction) in a total volume of 25 μl, and performed qPCR using Rotor-Gene Q (Qiagen). We amplified the GFP sequence and the Chinese hamster (CH) β-actin gene sequence for vector genome quantification and normalization, respectively. We used linearized plasmid containing the PCR target sequences as the vector genome copy number standards. The qPCR primer sequences are as follows:
GFP Forward: 5'-AGCAAAGACCCCAACGAGAA-3'
GFP Reverse: 5'-GGCGGCGGTCACGAA-3'
CHBA Forward: 5'-TGCGTGACATTAAGGAGAAG-3'
CHBA Reverse: 5'-CCAAGGAGGAGGAGGATG-3'

Plasmid construction

To construct pAAV9-SBBANN-AA-x-VBC-y and pAAV2R585E-SBBXEB-HP-x-VBC-y, we first constructed pAAV9-SBBANN-VBC-Lib and pAAV2R585E-SBBXEB-VBC-Lib, which are the backbone plasmid libraries carrying a pair (pr) of 12 nucleotide-long random DNA barcode sequences (pr-VBC). SBBANN and SBBXEB stand for 6 unique restriction enzyme recognition sites incorporated in each AAV viral genome (Sph I, Bsp EI, Bsi WI, Afl II, Nde I, and Nhe I for SBBANN; and Sph I, Bsp EI, Bsi WI, Xba I, Eag I, and Bgl II for SBBXEB). All of these plasmids carried the AAV2 *rep* gene, a *cap* gene derived from various AAV strains, the AAV2 polyadenylation signal (pA), DNA barcodes, and a 60-base pair (bp) qPCR target sequence derived from the open reading frame of the EGFP gene, in this order, between the two AAV2 inverted terminal repeats (ITRs).

We constructed pAAV9-SBBANN, based on the following parental plasmids; pUC620 (a wild-type AAV2 plasmid obtained from Avigen Inc.) and p5E18-VD2/9 (an AAV9 helper plasmid obtained from University of Pennsylvania). We replaced the AAV2 *cap* gene in pUC620 with the AAV9 *cap* gene in p5E18-VD2/9, and introduced the following modifications: a silent mutation T1818A in the AAV2 *rep* gene to create a unique Sph I site and silent mutations C546T, A549G, T1608C, G1611A, T1612A, C1613G and T1854A in the AAV9 *cap* gene (note: the numbers represent the nucleotide positions of each gene), which create unique Bsp EI, Afl II, and Nde I sites. Then we inserted a DNA fragment, (PBS1)-(Nhe I)-(PBS2/3)-(Bsr GI)-(PBS4-GFP), downstream of the pA. PBS1, PBS2/3 and PBS4 are 20-bp primer binding sequences; Nhe I and Bsr GI are 6-bp restriction enzyme recognition sites; and PBS4-GFP constitutes a 60-bp qPCR target. This procedure resulted in the construction of pAAV9-SBBANN. The presence of these unique restriction enzyme recognition sites facilitated site-directed mutagenesis of the AAV9 *cap* gene. We then created a double-stranded oligonucleotide containing a pair of 12 nucleotide-long random sequences and PBS2/3 by annealing and strand extension of the following two oligonucleotides:

67-62 For: 5'-CTAA<u>GCTAGC</u>NNNNNNNNNNNNACGGAAATACGATGTCGGGA-3'
67-62 Rev: 5'-TTCT**TGTACA**NNNNNNNNNNNNTCCCGACATCGTATTTCCGT-3'

The nucleotides indicated with an underline and bold-face letters are Nhe I and Bsr GI recognition sites, respectively. The annealed double-stranded oligonucleotide was then cut with Nhe I and Bsr GI and inserted between the unique Nhe I and Bsr GI recognition sites in pAAV9-SBBANN. This resulted in the creation of pAAV9-SBBANN-VBCLib, which had a (PBS1)-(Nhe I)-(lt-VBC)-(PBS2/3)-(rt-VBC)-(Bsr GI)-(PBS4-GFP) sequence downstream of pA. The lt- and rt-VBCs were 12 nucleotide-long random DNA sequences. The diversity of random DNA barcode sequences in pAAV9-SBBANN-VBCLib was 7.8×10^6.

High-throughput AA mutagenesis was performed as follows. We took an AA scanning strategy to analyze a wider region with a less number of mutants due to the proof-of-principle nature of the study. As shown in **Fig. 1e**, the construction of each mutant required 4 PCR primers (P1-For, P2-Rev, P3-For and P4-Rev). To scan the entire region between two restriction enzyme (RE) recognition sites RE1 and RE2 (*e.g.*, Bsi WI and Afl II recognition sites) by AA mutagenesis, we designed P1-For and P4-Rev common primers so that they can amplify a DNA segment spanning the entire RE1-RE2 region and containing at least 2 Dpn I recognition sites. We found that having at least 2 Dpn I sites is important for generating a high-quality library with a minimal background originating from the initial plasmid DNA template. We also designed each mutation-specific set of P2-Rev and P3-For primers. Although the lengths of P1-For and P4-Rev primers varied, P2-Rev and P3-For primers had a fixed length, 25 and 41 nucleotides, respectively. P2-Rev was complementary to the 25 nucleotides at the 5'-end of P3-For. The AA-

coding 6-nucleotide sequence (GCTGCT) was incorporated into the nucleotide positions 26-31 in P3-For. Except for this mismatched region, the nucleotide sequences in all the primers were completely matched to pAAV9-SBBANN. Using 10 ng of pAAV9-SBBANN DNA template and 0.25 unit of Platinum Pfx DNA Polymerase (Life Technologies), we PCR-amplified the P1-For/P2-Rev fragment and the P3-For/P4-Rev fragment separately in a 50 μl reaction mixture. PCR cycles for this amplification were 2 min at 95 °C, 35 cycles of 15 s at 95 °C, 30 s at 55 °C and 30-60 s at 68 °C, and subsequently 5 min at 68 °C. We then mixed the P1-For/P2-Rev and P3-For/P4-Rev PCR products together and treated them with 5 units of Dpn I (Roche Diagnostic) at 37 °C for 2 h in a total volume of 200 μl of reaction mixture in the presence of 1 × Buffer A (Roche Diagnostic). We purified the Dpn I-treated PCR products using QIAquick PCR Purification Kit (Qiagen), and performed a second PCR using one-fifth of the purified DNA, the P1-For and P4-Rev primers and 0.25 unit of Platinum Pfx DNA Polymerase. For this amplification, we used two-step PCR cycles as follows: 2 min at 95 °C, 35 cycles of 15 s at 95 °C, 30-60 s at 68 °C, and subsequently 5 min at 68 °C. We normally had PCR products for 10-50 mutants at a time. We then ran the PCR products on a 1.2% agarose gel along with DNA concentration standards, stained the gel with ethidium bromide, and quantified each PCR product by densitometry using Quantity One software (Bio-Rad). We proceeded to the following step using only a portion of each PCR product and stored the remaining at -20 °C for future use. We pooled up to 50 PCR products at an equimolar ratio, purified the mixture using QIAquick PCR Purification Kit, digested the eluted PCR products with a combination of restriction enzymes RE1 and RE2 (4 units per μg DNA for 1 h), ran them on a 1.2% agarose gel, purified them with QIAquick Gel Purification Kit (Qiagen), and quantified them by spectrophotometry. In the meantime, we digested 5 μg of pAAV9-SBBANN-VBCLib with 20 units each of RE1 and RE2 for 4 h followed by treatment of 20 units of Alkaline Phosphatase, Calf Intestinal (CIP, New England Biolabs (NEB)) at 37 °C for 1 h. We purified the resulting plasmid backbone DNA by gel electrophoresis and QIAquick Gel Purification Kit, and determined the quantity of DNA. We ligated 100 ng of the plasmid backbone and PCR products at a 1:3 molar ratio using T4 DNA Ligase (NEB) at 16 °C for at least 4 h in a total volume of 20 μl and transformed ElectroMax DH10B with 1 μl of the ligation mixture to make a site-directed mutant library on Luria Bertani (LB) agar in petri dishes. We then transferred the grown Escherichia coli colonies in the petri dishes to individual wells in 96-well LB agar plates, and shipped the plates to an outside sequencing service. We normally sequenced 4-5 times more colonies than the number of mutants contained in a library to obtain 2 to 3 clones carrying different VBCs per mutant. When we were not able to obtain all the clones we needed from a library, we repeated the procedure from the ligation step using the -20 °C-stored PCR products for the necessary mutants. Consequently, we were able to obtain all the pAAV9-SBBANN-AA-x-VBC-y plasmids used in the study.

pAAV2R585E-SBBXEB is the same as pUC118-AAV2R585E-SBBXEB-PBS[58] and was de novo synthesized. This plasmid has the AAV2 *rep* gene with the same silent mutation that creates an Sph I site, the following silent mutations in the AAV2R585E *cap* gene, and the

(PBS1)-(Nhe I)-(PBS2/3)-(Bsr GI)-(PBS4-GFP) DNA fragment downstream of the pA, between the two ITRs. The silent mutations introduced in the AAV2R585E *cap* gene are: G471C to create a unique Bsp EI site; C1167A to create a new Dpn I site; G1425A to create a unique Xba I site; C1638A to create a new Dpn I site; and C1776G and A1779C to create a new Eag I site (note: the numbers represent the nucleotide positions of the AAV2 *cap* gene). We also introduced a Bgl II site between the pA and PBS1. Consequently, pAAV2R585E-SBBXEB had the following 6 unique restriction enzyme recognition sites for site-directed mutagenesis; *i.e.*, SBBXEB. Then, we introduced a pair of 12 nucleotide-long random DNA sequences in the same manner as described above, and constructed pAAV2R585E-SBBXEB-VBCLib carrying random DNA barcodes with the diversity of 6.4×10^6.

High-throughput HP scanning mutagenesis was performed primarily in the same manner as that for AA mutagenesis as described earlier. We designed P1-For and P4-Rev common primers and each mutation-specific set of 25-mer P2-Rev and 53-mer P3-For primers. P2-Rev was complementary to the 5'-end 25 nucleotides of P3-For. Each HP-coding 18-nucleotide sequence was incorporated into the nucleotide positions 26-43 in P3-For. Except for this mismatched region, the nucleotide sequences in all the primers were completely matched to pAAV2R585E-SBBXEB. The subsequent steps for PCR, DNA ligation, bacterial transformation and sequencing were exactly the same as that for pAAV9-SBBANN-AA-x-VBC-y. As a result, we obtained all pAAV2R585E-SBBXEB-HP-x-VBC-y plasmids used in the study. We chose an HP scanning approach using AAV2R585E as a mutagenesis platform for the following reasons. AAV2R585E is a heparin binding-deficient AAV2 mutant that exhibits an infection-defective phenotype in many cell types[8]; therefore, we assumed that this mutant would provide an attractive platform for a mutagenesis study investigating gain-of-function phenotypes of heterologous HPs derived from other serotypes. Six consecutive amino acids (*i.e.*, HPs) were replaced because we assumed that only one or two amino-acid changes might not confer gain-of-function phenotypes.

We constructed pAAV-Serotype-x-VBC-y using pAAV9-SBBANN-VBCLib or pAAV2R585E-SBBXEB-VBCLib and a series of AAV helper plasmids. We replaced the AAV9 or AAV2R585E *cap* gene with that derived from various serotypes by restriction enzyme digestion followed by ligation.

Illumina sequencing

We treated approximately 5 μg of VBC-PCR amplicon mixture with T4 Polynucleotide Kinase (NEB), T4 DNA Polymerase (NEB) and DNA Polymerase I, Large (Klenow) Fragment (NEB) in the presence of 100 μM of dNTP and 1 mM ATP at 20 °C for 20 min. We then purified the end-repaired PCR products with QIAquick PCR Purification Kit, and treated them with Klenow Fragment (3′→5′ exo −) (NEB) in the presence of 500 μM of dATP to add adenine

bases to the 3' end of the PCR products. We again purified the PCR products with QIAquick MinElute PCR Purification Kit (Qiagen). We attached Y-shaped adaptors[62] to the ends of the PCR products with T4 DNA Ligase, and purified the resulting DNA with QIAquick PCR Purification Kit for the removal of free adaptors, which was followed by further purification by electrophoresis on a 3.0% agarose gel and QIAquick Gel Extraction Kit. We then amplified the purified DNA by four cycles of PCR with a set of adaptor-specific PCR primers and Platinum Pfx DNA Polymerase under the following PCR condition: 2 min at 95 °C, 4 cycles of 15 s at 95 °C, 30 s at 60 °C, and 30 s at 68 °C, and subsequently 5 min at 68 °C. We further purified the PCR products on a 3.0% agarose gel and sent 1-5 µg of DNA to Dragon Genomic Center, Takara Bio Inc. (Yokkaichi, Mie, Japan), where the samples were sequenced either with a 50 or 75-cycle single-end run on an Illumina Genome Analyzer IIx or with a 100-cycle single-end run on an Illumina HiSeq 2000.

Determination of the relative yields of virus production

We determined the relative viral particle production yield in the following manner. We produced AAVx_{ij} viral clones in separate culture dishes by DNA transfection and obtained crude cell lysates prepared in the same volume of the cell suspension buffer. We then pooled these crude cell lysates (10 µl each) and created the following 5 libraries: three AAV9-AA-VBCLib's (Lib-1, Lib-2 and Lib-3) and two AAV2R585E-HP-VBCLib's (Lib-1 and Lib-2) (**Table 1**). We extracted viral genome DNA from DNase I-resistant viral particles in each library, PCR-amplified lt- and rt-VBCs, Illumina-sequenced the PCR amplicons, and obtained Illumina sequence read number data for each VBC. We then adjusted the raw Illumina sequence read number data for each VBC by VBC-specific PCR amplification efficiency factor. The resulting adjusted Illumina sequence read numbers reflect relative viral particle production yield. The VBC-specific PCR amplification efficiency factor was determined in the following manner. We created an equimolar mixture of AAVx_{ij} plasmids used for the production of the 5 libraries described above. We created a total of 10 equimolar plasmid DNA mixtures (*i.e.*, 2 independent sets of 5 equimolar mixtures representing the 5 libraries), PCR-amplified lt- and rt-VBCs using the plasmid DNA mixture as templates, Illumina-sequenced the PCR amplicons, and obtained Illumina sequence read number data for each VBC. We then globally normalized Illumina sequence read numbers for each lt- and rt-VBC, which provides the PCR amplification efficiency factor for each VBC. To determine the relative viral particle production yield, we used the average of the two PCR amplification efficiency factors for the same VBC obtained from the 2 independent sets of the experiment.

4. SUPPLEMENTARY REFERENCE

62. Bentley, D.R. *et al.* Accurate whole human genome sequencing using reversible terminator chemistry. *Nature* **456**, 53-59 (2008).
